# Clinicopathological Significance of SCD Expression in Colon Adenocarcinoma: Association with Histological Grade and Exploratory Assessment in Relation to PCNA

**DOI:** 10.3390/jcm15176817

**Published:** 2026-09-02

**Authors:** Jerzy Z. Piecuch, Adam Piecuch, Marek Michalski, Magdalena Rynkiewicz, Natalia Matysiak, Magdalena Onyszczuk, Marlena Brzozowa-Zasada

**Affiliations:** 1 Department of General and Bariatric Surgery and Emergency Medicine in Zabrze, Faculty of Medical Sciences in Zabrze, Medical University of Silesia, 40-055 Katowice, Poland; 2 Department of Histology and Cell Pathology in Zabrze, Faculty of Medical Sciences in Zabrze, Medical University of Silesia in Katowice, 40-055 Katowice, Poland; mmichalski@sum.edu.pl (M.M.); nmatysiak@sum.edu.pl (N.M.); mbrzozowa@sum.edu.pl (M.B.-Z.); 3 Department of Pathomorphology, Faculty of Medical Sciences in Zabrze, Medical University of Silesia, 40-055 Katowice, Poland; mrynkiewicz@sum.edu.pl (M.R.); monyszczuk@sum.edu.pl (M.O.)

**Keywords:** stearoyl-CoA desaturase, SCD, colon adenocarcinoma, PCNA, lipid metabolism, immunohistochemistry, exploratory combined index

## Abstract

**Background/Objectives:** Colon adenocarcinoma is a heterogeneous disease, and tumours of similar pathological stage may differ in their metabolic and proliferative characteristics. Stearoyl-CoA desaturase (SCD) is involved in lipid metabolic reprogramming and may reflect aspects of tumour biology. The main goal of this study was to analyse SCD expression and its associations with clinicopathological factors, particularly focusing on histological grade. The relationship between SCD and proliferating cell nuclear antigen (PCNA) was evaluated as a secondary objective. **Methods:** Public TCGA/UALCAN and CPTAC/UALCAN data were examined as supportive transcriptomic and proteomic observations. Immunohistochemical SCD and PCNA expression was assessed in 113 patients with colon adenocarcinoma, and SCD localisation was additionally examined by qualitative immunogold electron microscopy. Standardised SCD and PCNA scores were combined with equal weighting to generate a secondary exploratory z-score. Associations with clinicopathological characteristics were assessed using appropriate univariable analyses, while the relationship between SCD and PCNA and the potential incremental contribution of SCD beyond PCNA were examined using correlation and exploratory regression-based analyses. ROC analyses were used only as exploratory descriptions of statistical separation between predefined clinicopathological groups. **Results:** Observations from supportive public databases indicated that SCD expression was higher in tumour tissue compared to normal tissue at both the transcriptomic and proteomic levels. In the institutional cohort, SCD expression was elevated in tumour tissue relative to matched non-neoplastic mucosa and was associated primarily with histological grade but not with nodal status or pathological stage. SCD showed a modest positive correlation with PCNA. The secondary exploratory combined SCD–PCNA score was associated with histological grade and with nodal status but did not outperform PCNA alone. Adding SCD to PCNA provided only limited incremental information for G3 histology and no incremental value for nodal involvement. **Conclusions:** SCD is a tumour-associated metabolic marker in colon adenocarcinoma, with its strongest association observed for histological grade. The combined score should not be interpreted as a biological entity or superior biomarker.

## 1. Introduction

Colon adenocarcinoma continues to be one of the most prevalent cancers globally and a significant contributor to cancer-related deaths. Although tumour-node-metastasis (TNM) staging remains the foundation of clinical decision-making, tumours classified within the same anatomical stage may differ in biological behaviour, metastatic potential, and treatment response [1]. This heterogeneity is relevant in intermediate categories of disease, where conventional histopathological parameters do not always fully reflect the underlying molecular and metabolic phenotype of the tumour. Modern molecular classifications have further demonstrated that colorectal cancer comprises biologically distinct subgroups with different clinical and therapeutic implications [2,3]. Therefore, there is a continuing need to identify tissue-based markers that complement routine morphology and provide additional insights into tumour biology.

Metabolic reprogramming has now been established as a key characteristic of cancer. Beyond altered glucose metabolism, malignant cells frequently alter the metabolism of lipids to support biosynthesis of cell membranes, redox balance, energy storage, signal transduction, and adaptation to cellular stress [4,5,6,7]. Tumour cells that proliferate rapidly require a continuous supply of lipids in order to support cell membrane biogenesis. At the same time, changes in lipid saturation influence membrane fluidity, endoplasmic reticulum stress, lipotoxicity, and oncogenic signalling. These observations have drawn attention to enzymes involved in fatty acid synthesis and desaturation as potential mediators of tumour progression and as candidate biomarkers of aggressive tumour biology.

Stearoyl-CoA desaturase (SCD), also referred to as SCD1 in many mechanistic studies, serves as a pivotal enzyme in lipid metabolic pathways. It mediates the conversion of saturated fatty acids into monounsaturated ones, mainly oleic and palmitoleic acid [8,9,10]. Through this reaction, SCD regulates the balance between saturated and unsaturated lipid species and contributes to the synthesis of phospholipids, triglycerides, cholesterol esters, and other lipid components essential for cellular homeostasis and membrane integrity. In cancer cells, increased SCD activity may help maintain membrane plasticity, support proliferation, and protect cells from saturated fatty acid-induced lipotoxic stress [9,11]. Experimental studies in several tumour models have suggested that SCD1 may promote cancer cell survival and that its inhibition can impair tumour cell growth, highlighting the potential biological relevance of this enzyme in malignancy [11,12]. In colorectal cancer models, SCD1 has also been linked to apoptosis regulation and ceramide-related lipid metabolism [13].

Previous studies have already demonstrated increased SCD1 expression and biological relevance in colorectal cancer. Ran et al. reported increased SCD1 expression in colorectal cancer and implicated SCD1 in metastatic progression, including glucose-responsive regulation of PTEN/Akt signalling [14]. Wu et al. subsequently reported increased SCD1 expression in paired human colorectal cancer tissues and linked SCD1-mediated lipid desaturation to metastatic behaviour and ferroptosis resistance [15]. More recently, Wang et al. provided an integrated analysis of SCD expression and its clinicopathological significance in colorectal cancer using multicentre high-throughput data together with immunohistochemical and functional analyses [16]. Thus, tumour-associated upregulation of SCD itself should be regarded as an established observation rather than a novel finding. However, the relationship between SCD expression and histological differentiation, its association with a proliferation-related marker such as PCNA, and its ultrastructural localisation in human colon adenocarcinoma tissue remain less well characterised.

Another relevant process is proliferation. PCNA is an essential component of DNA replication and repair and has been widely used as an immunohistochemical marker of proliferative activity [17,18]. Because proliferating tumour cells have increased biosynthetic and lipid requirements, examining SCD in relation to PCNA may provide biological context linking lipid metabolism and proliferative activity. PCNA was selected in the pre-sent study as a proliferation-associated marker because of its direct involvement in DNA replication and repair and its established use in immunohistochemical assessment of proliferative activity. Its role here was to provide biological proliferative context for SCD ex-pression rather than to represent a clinically preferred proliferation marker; Ki-67 is more widely used in routine pathological practice.

Histological grade, tumour budding, and tumour-infiltrating lymphocytes provide complementary information on tumour differentiation, invasiveness, and the tumour microenvironment [19]. Assessing SCD in relation to these clinicopathological characteristics may therefore help define its biological context within colon adenocarcinoma without implying a validated diagnostic or prognostic application.

Accordingly, the present study was designed not to establish tumour-associated SCD upregulation as a novel observation but to extend the existing evidence by examining its clinicopathological context in colon adenocarcinoma. Public TCGA/UALCAN and CPTAC/UALCAN data were examined as supportive observations to assess whether tumour-associated SCD upregulation was also evident at the transcriptomic and proteomic levels. SCD protein expression was then assessed immunohistochemically in an institutional cohort and compared between tumour tissue and non-neoplastic colonic mucosa. Ultrastructural immunogold labelling was used as a supportive, descriptive approach to examine the intracellular localisation of SCD immunoreactivity rather than to establish a novel subcellular localisation pattern. Associations between SCD expression and clinicopathological parameters were also examined. Finally, because lipid metabolism and proliferation are biologically interconnected, SCD expression was analysed in relation to PCNA, while the combined SCD–PCNA z-score was treated as a secondary exploratory metabolic–proliferative index.

## 2. Materials and Methods

### 2.1. Supportive Public-Database Analyses of SCD Expression

#### 2.1.1. Supportive TCGA/UALCAN Transcriptomic Analysis

Colon adenocarcinoma transcriptomic data were collected from The Cancer Genome Atlas (TCGA) via the UALCAN interactive web platform (http://ualcan.path.uab.edu) [20,21], accessed on 25 and 26 June 2026. SCD mRNA expression was analysed using the TCGA colon adenocarcinoma (COAD) dataset available through UALCAN. The comparison between sample types included 286 primary colon adenocarcinoma samples and 41 normal colonic tissue samples. Gene expression values were reported as transcripts per million (TPM).

**Figure 1:**
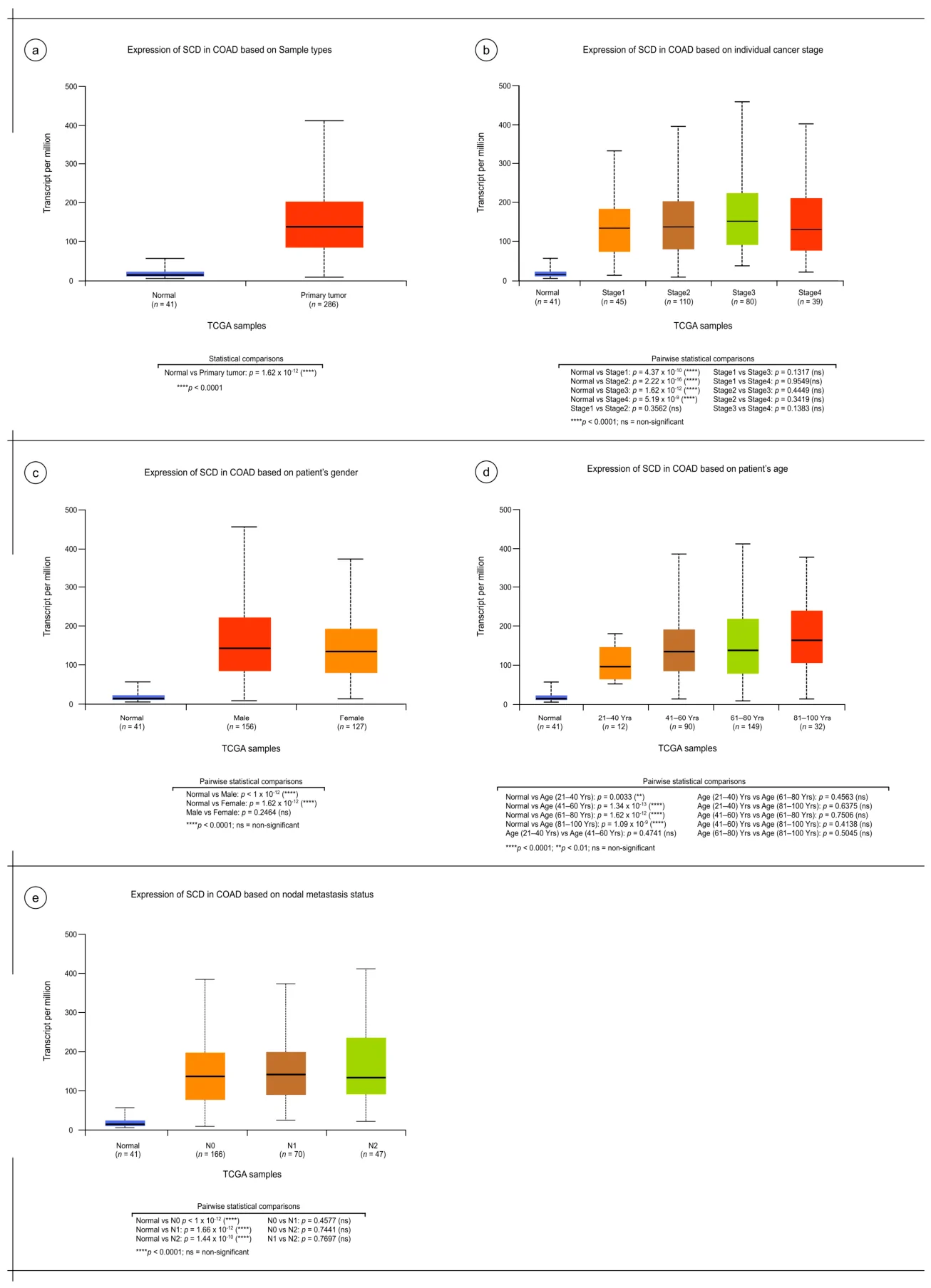
Supportive TCGA/UALCAN observations of SCD mRNA expression in colon adenocarcinoma. (**a**) Comparison of SCD mRNA expression between normal colonic tissue (*n* = 41) and primary colon adenocarcinoma (*n* = 286). (**b**) SCD expression according to pathological stage. (**c**) SCD expression according to patient sex. (**d**) SCD expression according to age category. (**e**) SCD expression according to nodal metastasis status. SCD mRNA expression was significantly higher in tumour samples than in normal tissue across all analysed clinicopathological subgroups. No significant differences were observed between individual tumour stages; between male and female patients; between age categories; or between N0, N1, and N2 groups. These UALCAN-based comparisons are presented as supportive public-database observations. Expression values are presented as transcripts per million. Boxplots show the median and interquartile range, with whiskers indicating the data range. Pairwise *p* values are shown below each panel. Abbreviations: COAD, colon adenocarcinoma; SCD, stearoyl-CoA desaturase; TCGA, The Cancer Genome Atlas; TPM, transcripts per million; ns, not significant.

SCD mRNA expression was compared between normal colonic tissue and primary tumour samples. Additional subgroup analyses were performed according to pathological stage, sex, age category, and lymph node metastasis status, based on the subgroup definitions available within UALCAN. Statistical significance for pairwise comparisons was assessed using the unpaired two-sample *t*-test implemented in UALCAN. A two-sided *p* value < 0.05 was considered statistically significant. No additional multiplicity correction was applied by the authors to the platform-derived subgroup *p* values; these analyses were therefore treated as descriptive and supportive rather than confirmatory. No additional patient-level statistical modelling was performed using the TCGA/UALCAN data. The platform-generated plots and pairwise *p* values used for interpretation are reproduced in Figure 1.

#### 2.1.2. Supportive CPTAC/UALCAN Proteomic Analysis

Protein expression data were obtained from the Clinical Proteomic Tumor Analysis Consortium (CPTAC) colon cancer cohort through the UALCAN platform (http://ualcan.path.uab.edu), accessed on 25 and 26 June 2026. The sample-type comparison comprised 97 primary tumour samples and 100 normal tissue samples. SCD protein abundance was expressed as Z-values using the data representation provided by UALCAN.

**Figure 2:**
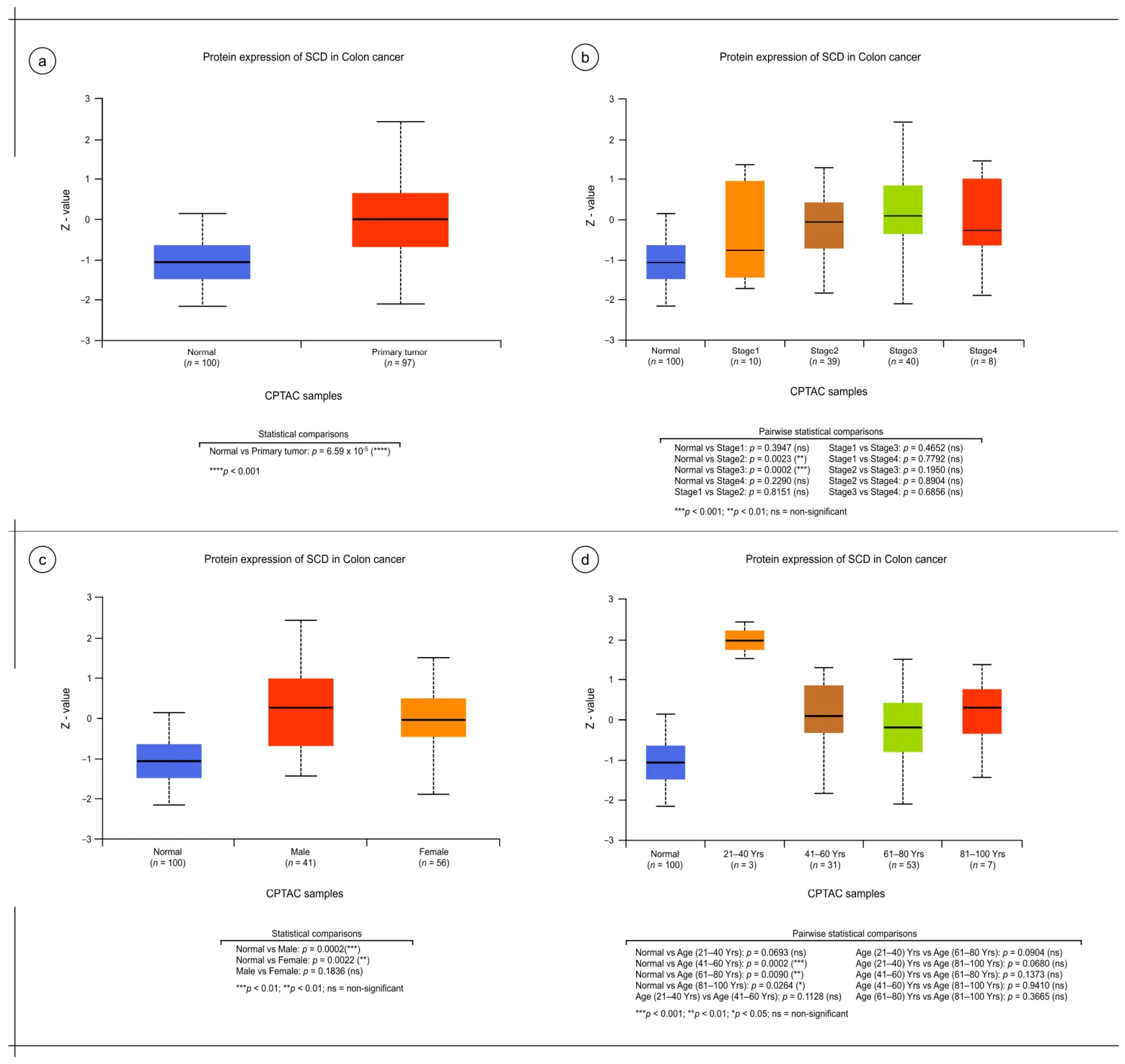
Supportive CPTAC/UALCAN observations of SCD protein expression in colon cancer. (**a**) Comparison of SCD protein expression between normal tissue (*n* = 100) and primary colon cancer (*n* = 97). (**b**) SCD protein expression according to pathological stage. (**c**) SCD protein expression according to patient sex. (**d**) SCD protein expression according to age category. SCD protein abundance was significantly higher in primary tumour samples than in normal tissue. In stage-stratified analyses, significant differences relative to normal tissue were observed for stage II and stage III tumours but not for stage I or stage IV tumours. SCD protein expression was also significantly higher than in normal tissue in both male and female patients and in the 41–60-, 61–80-, and 81–100-year age groups. No significant differences were observed between individual tumour stages, between sexes, or between age categories. These UALCAN-based comparisons are presented as supportive public-database observations. Protein abundance values are presented as Z-values as displayed by UALCAN. Pairwise *p* values are shown below each panel. Abbreviations: CPTAC, Clinical Proteomic Tumour Analysis Consortium; SCD, stearoyl-CoA desaturase; ns, not significant.

SCD protein expression was compared between normal colonic tissue and primary colon cancer samples. Additional subgroup analyses were performed according to pathological stage, sex, and age category, based on the subgroup definitions available within UALCAN. Pairwise statistical comparisons were performed using the unpaired two-sample *t*-test implemented in UALCAN. A two-sided *p* value < 0.05 was considered statistically significant. No additional multiplicity correction was applied by the authors to the platform-derived subgroup *p* values; these analyses were therefore treated as descriptive and supportive rather than confirmatory. No additional patient-level statistical modelling was performed using the CPTAC/UALCAN data. The platform-generated plots and pairwise *p* values used for interpretation are reproduced in Figure 2.

The TCGA/UALCAN and CPTAC/UALCAN components were used only as supportive public-database observations to assess whether tumour-associated SCD upregulation was also evident at the transcriptomic and proteomic levels. These analyses were limited to aggregated platform-based comparisons and were not intended as patient-level, multivariable, survival, or independent validation analyses of the institutional findings, the SCD–PCNA combined score, or its clinicopathological performance.

### 2.2. Immunohistochemistry

This retrospective study used archival cases of histologically confirmed colon adenocarcinoma from patients who underwent surgical resection between 2018 and 2023 at the Department of General and Bariatric Surgery and Emergency Medicine in Zabrze, Faculty of Medical Sciences in Zabrze, Medical University of Silesia. Archival anonymised formalin-fixed, paraffin-embedded (FFPE) tissue blocks together with corresponding anonymised clinicopathological data were retrieved from the archive of the Department of Pathomorphology. No prospective patient recruitment was performed.

The institutional cohort was restricted to patients with pathological stage II or III colon adenocarcinoma. Stage I and stage IV cases were excluded because they were only sparsely represented in the available archival material and their small numbers would not have permitted reliable subgroup analyses. A total of 150 archival cases were initially assessed, of which 37 were excluded for one or more predefined reasons, resulting in a final analytical cohort of 113 patients. The exclusion criteria included neoadjuvant treatment, hereditary cancer syndrome, synchronous malignancy, inflammatory bowel disease, insufficient quantity or quality of archival tissue material, and pathological stage I or IV disease.

Histological grade, tumour-infiltrating lymphocyte density, and tumour budding were obtained retrospectively from the anonymised archival histopathology reports and were not re-assessed specifically for the present study. These variables were analysed according to the categories recorded in the original pathology documentation. Pathological staging and histological grading were therefore based on the original diagnostic reports and were not retrospectively reassigned for the purposes of the present study. Tumour sidedness was classified according to anatomical location. Right-sided colon cancers included tumours of the caecum, ascending colon, hepatic flexure, and transverse colon, whereas left-sided colon cancers included tumours of the splenic flexure, descending colon, and sigmoid colon.

Archival FFPE tissue blocks of colon adenocarcinoma and corresponding patient-matched non-neoplastic colonic mucosa were selected for immunohistochemical analysis. For each of the 113 patients, tumour tissue and a paired non-neoplastic mucosal sample were available. Non-neoplastic mucosa was obtained from a macroscopically unchanged area of the same resected specimen, located at least 5 cm from the tumour, and was histologically confirmed to be free of neoplastic or dysplastic lesions. Tumour tissue and the corresponding non-neoplastic colonic mucosa were scored separately.

FFPE blocks were sectioned at a thickness of 4 µm, mounted on Polysine-coated slides, deparaffinized in xylene, and rehydrated through a descending ethanol gradient. Heat-induced epitope retrieval was performed in 10 mM of citrate buffer (pH 6.0), utilising microwave heating for two consecutive 8 min cycles. After cooling and washing, the sections were incubated with primary antibodies against SCD (1:600; GeneTex 77829) and PCNA (1:1000; Zytomed Systems, Berlin, Germany; REF 516-2850) under optimised conditions at 4 °C for 24 h. Target protein visualisation was achieved using the BrightVision detection system with Permanent AP Red Chromogen, followed by nuclear counterstaining with Mayer’s haematoxylin.

Immunohistochemical staining was evaluated using a modified immunoreactive score (IRS), based on staining intensity and the percentage of positive cells. Staining intensity was graded as follows: 0, no staining; 1, weak staining; 2, moderate staining; and 3, strong staining. The percentage of positive cells was scored as follows: 0, no positive cells; 1, 1–25%; 2, 26–50%; 3, 51–75%; and 4, 76–100% [22]. The final IRS value ranged from 0 to 12 and was calculated by multiplying the staining intensity score by the percentage score.

For SCD, only unequivocal cytoplasmic and/or membranous-cytoplasmic staining in viable epithelial or tumour cells was considered positive. For PCNA, only nuclear staining in viable epithelial or tumour cells was considered positive. Stromal staining, inflammatory cells, necrotic areas, mucin pools, luminal debris, tissue folds, edge artefacts, and nonspecific background staining were excluded from IRS evaluation. For PCNA, the IRS approach was used as a semi-quantitative measure of nuclear immunoreactivity, incorporating both the proportion of positively stained tumour nuclei and staining intensity. This approach was selected to provide a scoring framework consistent with that used for SCD and to facilitate within-cohort analyses of the relationship between the two markers. PCNA IRS was not intended to represent a conventional labelling index or a clinically validated proliferation cut-off.

Immunohistochemical scoring was performed on whole-tissue sections. The entire slide was initially examined at low magnification. Four viable invasive tumour fields were then selected using a 20× objective to reflect the overall range of staining intensities observed across the section rather than preferentially sampling areas with the strongest staining. Corresponding non-neoplastic colonic mucosa was evaluated separately as a reference tissue compartment and was excluded from the tumour IRS evaluation. The immunohistochemical analysis included 113 patients with complete paired SCD IRS measurements in colon adenocarcinoma tissue and corresponding non-neoplastic colonic mucosa, as well as complete tumour PCNA IRS data.

Histological and immunohistochemical slide evaluations were conducted independently by two experienced observers who were blinded to the patient clinicopathological data during scoring. To account for intratumoural staining heterogeneity, the mean expression across the four selected tumour fields was used for the final tumour-level IRS assessment. When discrepant assessments occurred between the two observers, the respective areas were jointly re-evaluated, and a consensus final score was established for subsequent analysis. Observer-specific IRS values were not retained; therefore, a formal quantitative measure of interobserver agreement could not be calculated retrospectively. Negative controls were included to assess nonspecific staining. Formal repeat-staining experiments or technical replicate analyses were not performed; therefore, staining reproducibility was not quantitatively assessed.

### 2.3. Immunogold Transmission Electron Microscopy

For ultrastructural localisation of SCD, tissue samples were fixed in 4% paraformaldehyde prepared in 0.1 M of phosphate-buffered saline (PBS) for 2 h at room temperature. Samples were then rinsed in PBS, dehydrated through a graded ethanol series, and embedded in LR White resin. Ultrathin sections, approximately 70 nm thick, were cut using an RMC Boeckeler PowerTome PC ultramicrotome equipped with a diamond knife (Diatome AG, Nidau, Switzerland) and mounted on Formvar-coated nickel grids.

The sections were pre-incubated in 50 mM of NH_4_Cl for 30 min and blocked with 1% bovine serum albumin (BSA) in PBS for 30 min. The grids were subsequently incubated overnight at 4 °C with a rabbit anti-SCD primary antibody (GeneTex 77829; dilution 1:50). Immunogold detection was performed using a 15 nm gold-conjugated goat anti-rabbit IgG secondary antibody (BBI Solutions, Cardiff, UK; 1:100) for 1 h at room temperature.

After washing, the sections were contrasted with 0.5% uranyl acetate. Negative controls were processed in parallel under identical conditions, except that the primary antibody was omitted.

The grids were examined using a Tecnai 12 G2 Spirit BioTWIN transmission electron microscope (FEI Company, Hillsboro, OR, USA) operated at 120 kV. Images were acquired using a Morada CCD camera.

The immunogold TEM component was performed on archival tissue material from 10 patients and was designed as a qualitative, descriptive localisation analysis. The ultrastructural assessment focused on the presence and subcellular distribution of SCD-associated immunogold labelling. No formal gold-particle counting or particle-density measurements were performed. Negative-control preparations were processed with omission of the primary antibody to assess nonspecific labelling. Antibody-related control assessment was limited to this negative control; no additional orthogonal antibody-validation procedures or repeat-staining reproducibility experiments were performed. Accordingly, the TEM component was intended as a descriptive and supportive ultrastructural analysis rather than as a quantitative analytical endpoint.

### 2.4. Statistical Analysis

Statistical analyses were performed using Statistica version 9.1 and PQStat version 1.8.2.

Categorical variables are presented as numbers and percentages. Continuous variables were summarised using the mean ± standard deviation (SD), median with interquartile range [Q1–Q3], and minimum–maximum values.

The immunohistochemical expression of SCD and PCNA was assessed using immunoreactive score (IRS) values. Because IRS values are semi-quantitative ordinal measures, non-parametric methods were used for their primary analyses. SCD IRS values in colon adenocarcinoma tissue and corresponding non-neoplastic mucosa were compared in 113 patient-matched pairs using the two-sided Wilcoxon signed-rank test. Comparisons be-tween two independent groups were performed using the Mann–Whitney U test, whereas comparisons among more than two groups were performed using the Kruskal–Wallis test. When the overall Kruskal–Wallis test was statistically significant, post hoc pairwise comparisons were conducted. Associations between IRS values and ordinal clinicopathological variables were assessed using Spearman’s rank correlation coefficient. The relationship between SCD IRS and PCNA IRS was evaluated using the same method. For IRS analyses, post hoc pairwise comparisons following a significant Kruskal–Wallis test were performed using Dunn’s test with Holm adjustment for the pairwise comparisons within that family.

To explore the joint expression of the metabolic and proliferative markers, standardised z-scores were calculated separately for SCD IRS and PCNA IRS. The combined SCD–PCNA z-score was defined as the sum of the two standardised values, thereby assigning equal weight to both markers. This deliberately simple, sample-dependent composite was used exclusively for hypothesis-generating analyses and was not regarded as an empirically optimised model, a distinct biological classification, or a clinically validated biomarker.

For analyses of the combined SCD–PCNA z-score, comparisons between two groups were performed using Student’s *t*-test when parametric assumptions were satisfied and Welch’s *t*-test when variances were unequal. Comparisons among more than two groups were performed using one-way analysis of variance, followed by post hoc pairwise com-parisons when appropriate. Associations with ordinal clinicopathological variables were assessed using Spearman’s rank correlation coefficient. Following a significant one-way ANOVA, pairwise comparisons were performed using Tukey’s honestly significant difference (HSD) test.

A secondary sensitivity analysis examined the association between the combined SCD–PCNA z-score and poorly differentiated G3 histology using logistic regression. Be-cause the composite score was sample-dependent, equally weighted, and not externally validated, this analysis was considered hypothesis-generating and was not used as evidence of incremental value beyond PCNA. Full model specifications and results are provided in Supplementary Material S2 (Methods and Supplementary Table S1).

To determine whether SCD provided information beyond PCNA, separate nested logistic regression models were fitted for each binary endpoint. Model A included PCNA IRS alone, whereas Model B included PCNA IRS and SCD IRS as separate continuous predictors. The incremental contribution of SCD was evaluated using the likelihood-ratio test, changes in Akaike information criterion (AIC) and McFadden pseudo-R^2^, and the adjusted OR for SCD after accounting for PCNA. This nested-model comparison was considered the principal analysis of incremental value. The combined z-score was not used as a substitute for directly testing whether SCD improved a model already containing PCNA.

For logistic regression models in which SCD IRS and PCNA IRS were entered as continuous predictors, linearity in the logit was assessed using Box–Tidwell-type predictor-by-log-transformed-predictor terms.

For the histological-grade endpoint, ROC analysis was used solely as an exploratory description of statistical separation between tumours with already established G3 histology and G1/G2 tumours. Because histological grade had been determined by routine pathological examination of the same resection specimens, this analysis was not intended to assess diagnostic accuracy, reclassification, or clinical utility. AUCs with 95% CIs were retained in the main analysis as descriptive measures of separation; Youden-derived cut-offs, sensitivity, and specificity for the grade-based ROC analysis are reported only in Supplementary Material S2, Table S2. Pairwise AUC comparisons were performed using the DeLong method. Youden-derived cut-offs, sensitivities, and specificities were calculated and evaluated in the same cohort and should therefore be regarded as apparent, exploratory estimates. These threshold-based measures were not internally or independently validated.

All statistical tests were two-sided, and *p* values < 0.05 were considered statistically significant. Borderline findings were interpreted cautiously. Analyses involving the combined SCD–PCNA z-score were regarded as secondary, exploratory, and hypothesis-generating. No assumption was made that the score represented a distinct biological entity or a clinically validated biomarker.

Given the exploratory nature of the secondary and subgroup analyses and the number of clinicopathological comparisons performed, no formal adjustment for multiple testing was applied. Accordingly, *p* values from secondary and subgroup analyses should be regarded as nominal and interpreted cautiously, together with effect estimates and confidence intervals, rather than as definitive confirmatory evidence. These analyses were intended primarily to identify patterns warranting confirmation in larger independent cohorts.

The institutional analyses used complete-case data. All 113 included patients had complete values for SCD IRS, PCNA IRS, histological grade, pT, pN, pathological stage, age, sex, tumour location, tumour budding, tumour-infiltrating lymphocyte density. No imputation of missing data was performed.

No a priori sample-size calculation was performed because this was a retrospective analysis of the available institutional cohort. The final cohort comprised 113 patients, including 24 G3 tumours. Given the limited number of G3 events relative to the number of potential predictors, multivariable logistic regression models were fitted with a limited number of prespecified covariates, and effect estimates, confidence intervals, and consistency across analyses were emphasised over isolated borderline *p* values.

## 3. Results

### 3.1. Supportive TCGA/UALCAN Observations of SCD mRNA Expression

The supportive UALCAN-based analysis of TCGA data showed higher SCD mRNA expression in primary colon adenocarcinoma than in normal colonic tissue (*p* = 1.62 × 10^−12^; Figure 1a). Similar tumour-associated elevation was observed across the available clinicopathological subgroups, whereas no significant differences were observed between tumour stages, sexes, age categories, or nodal categories (Figure 1b–e).

### 3.2. Supportive CPTAC/UALCAN Observations of SCD Protein Expression

The supportive UALCAN-based analysis of CPTAC data showed higher SCD protein expression in primary colon cancer than in normal colonic tissue (*p* = 6.59 × 10^−5^; Figure 2a). Subgroup comparisons were broadly consistent with tumour-associated elevation, while no significant differences were observed between individual tumour stages, sexes, or age categories (Figure 2b–d).

### 3.3. Clinicopathological Characteristics of the Institutional Cohort

The institutional cohort included 113 patients with colon adenocarcinoma. Male patients accounted for 56.6% of the cohort and female patients for 43.4%. The mean age was 66.6 ± 9.2 years, with a median of 66 years (Table 1).

Moderately differentiated tumours represented the dominant histological category. Histological grade distribution included 11 G1 tumours, 78 G2 tumours, and 24 G3 tumours. Most tumours were locally advanced, with pT3 tumours comprising the largest subgroup. Lymph node involvement was present in 55 patients, whereas 58 patients were node-negative. The cohort included only stage II and stage III tumours; consequently, pathological stage was determined by nodal status, with all pN0 tumours classified as stage II and all pN1/pN2 tumours as stage III. Analyses of pathological stage and nodal status therefore represented the same binary division of the cohort and should not be interpreted as independent findings (Table 1).

Low tumour-infiltrating lymphocyte density was the most frequent immune pattern. Tumour budding was most commonly classified as Bd1 or Bd2, whereas Bd3 tumours were less frequent (Table 1).

### 3.4. SCD Expression Is Elevated in Tumour Tissue and Is Associated Mainly with Histological Grade

**Figure 3:**
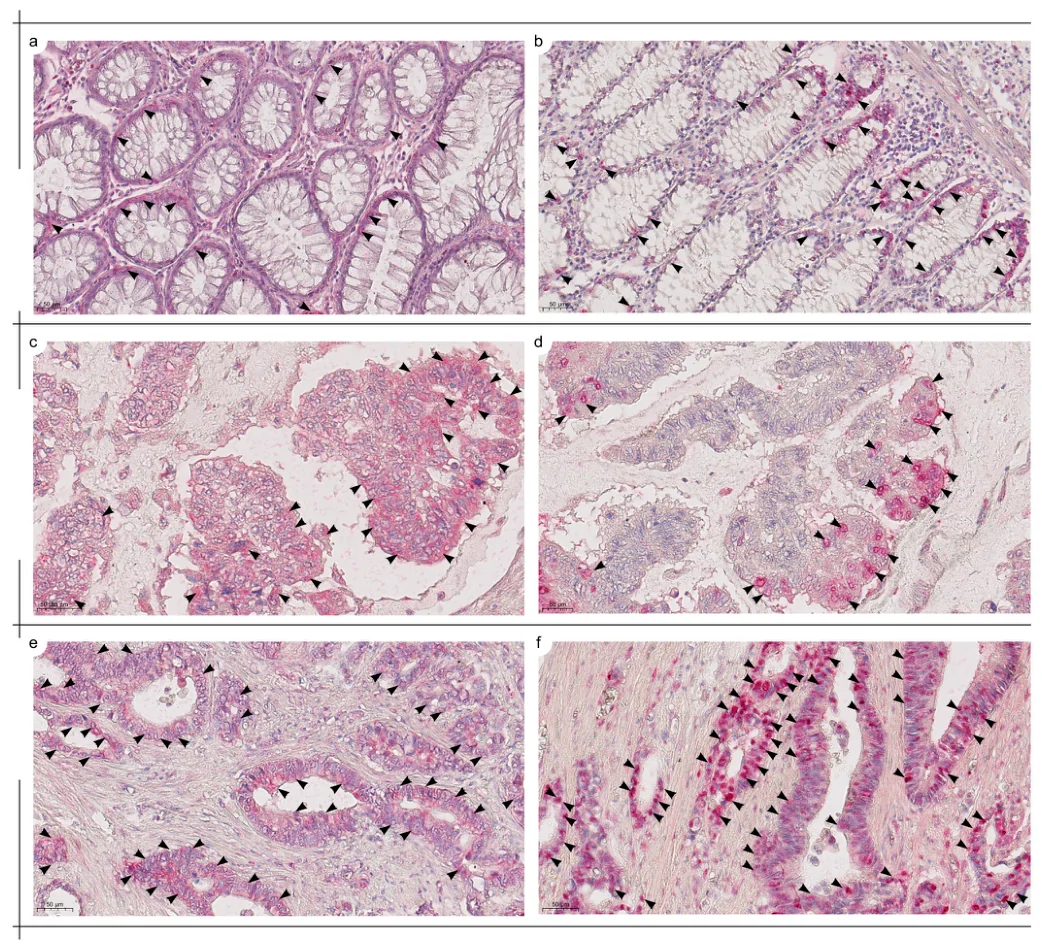
Representative immunohistochemical staining of SCD and PCNA in non-neoplastic colonic mucosa and colon adenocarcinoma tissue. Panels (**a**,**b**) show non-neoplastic colonic mucosa, whereas panels (**c**–**f**) show colon adenocarcinoma tissue. Panels (**a**,**c**,**e**) show SCD immunostaining, and panels (**b**,**d**,**f**) show PCNA immunostaining. Panels (**c**,**d**) represent SCD and PCNA immunostaining from one representative colon adenocarcinoma case, whereas panels e and f represent SCD and PCNA immunostaining from a second representative case. SCD immunoreactivity is predominantly cytoplasmic, whereas PCNA immunoreactivity is predominantly nuclear. Positive immunoreactivity is visualised as a red reaction product generated by Permanent AP Red Chromogen, with nuclear counterstaining performed using Mayer’s haematoxylin. Arrowheads indicate representative positively stained epithelial or tumour cells. Scale bars: 50 µm. Negative control sections, processed without the primary antibody, are shown in Supplementary Material S1, Figure S1, and exhibit no significant nonspecific staining.

Immunohistochemical analysis showed significantly higher SCD expression in colon adenocarcinoma tissue than in the corresponding patient-matched non-neoplastic colonic mucosa (Figure 3). Tumour samples demonstrated a mean SCD IRS value of 5.45 ± 2.29 and a median value of 5 [Q1–Q3: 4–7], whereas the paired non-neoplastic mucosal samples showed a mean IRS of 1.79 ± 0.95 and a median value of 2 [Q1–Q3: 1–2]. In the 113 paired observations, tumour IRS exceeded mucosal IRS in 107 patients and was equal in 6 patients; no patient had a lower tumour IRS. The two-sided Wilcoxon signed-rank test confirmed a highly significant paired difference (W = 0; Z = −9.006; *p* < 0.001; Table 2).

### 3.5. Subcellular Localisation of SCD by Immunogold Labelling and Transmission Electron Microscopy

Immunogold labelling using transmission electron microscopy (TEM) was performed as a complementary ultrastructural localisation study to evaluate the subcellular distribution of SCD immunoreactivity in colon adenocarcinoma tissue. This analysis aimed to show the presence and intracellular localisation of SCD labelling rather than to quantify SCD expression levels.

**Figure 4:**
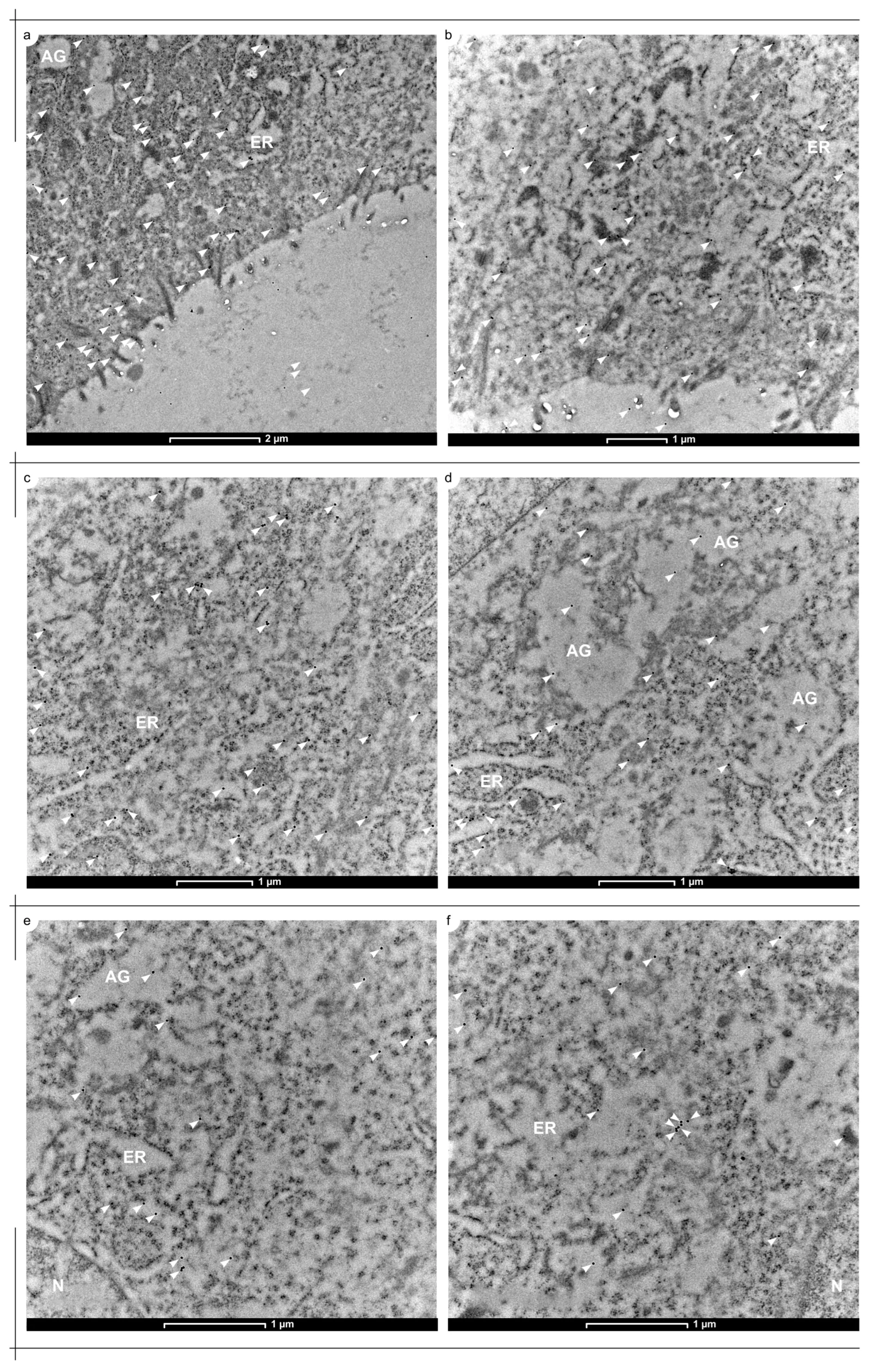
Immunogold-based subcellular localisation of SCD protein in non-neoplastic colonic mucosa and colon adenocarcinoma tissue using transmission electron microscopy. Panels (**a**,**b**) show non-neoplastic colonic mucosa, whereas panels (**c**–**f**) show colon adenocarcinoma tissue. Gold particles indicating SCD labelling are visible mainly within the cytoplasmic compartment and in association with membranous structures morphologically compatible with the endoplasmic reticulum. Occasional labelling is also seen near membranous compartments compatible with the Golgi apparatus. Arrowheads indicate representative immunogold particles. ER, endoplasmic reticulum; AG, Golgi apparatus; N, nucleus. Scale bars: 1–2 µm, as indicated.

SCD immunogold labelling was observed predominantly within the cytoplasmic compartment of tumour cells. Gold particles were mainly associated with intracellular membranous structures morphologically compatible with the endoplasmic reticulum. Labelling was observed along endoplasmic reticulum-like membrane profiles and in the perinuclear cytoplasm. Occasional immunogold particles were also seen close to membranous compartments compatible with the Golgi apparatus. Nuclear labelling was not a characteristic feature (Figure 4). These observations support a predominantly cytoplasmic, membrane-associated pattern of SCD immunoreactivity, while this TEM analysis was descriptive and not intended for quantitative assessment.

**Figure 5:**
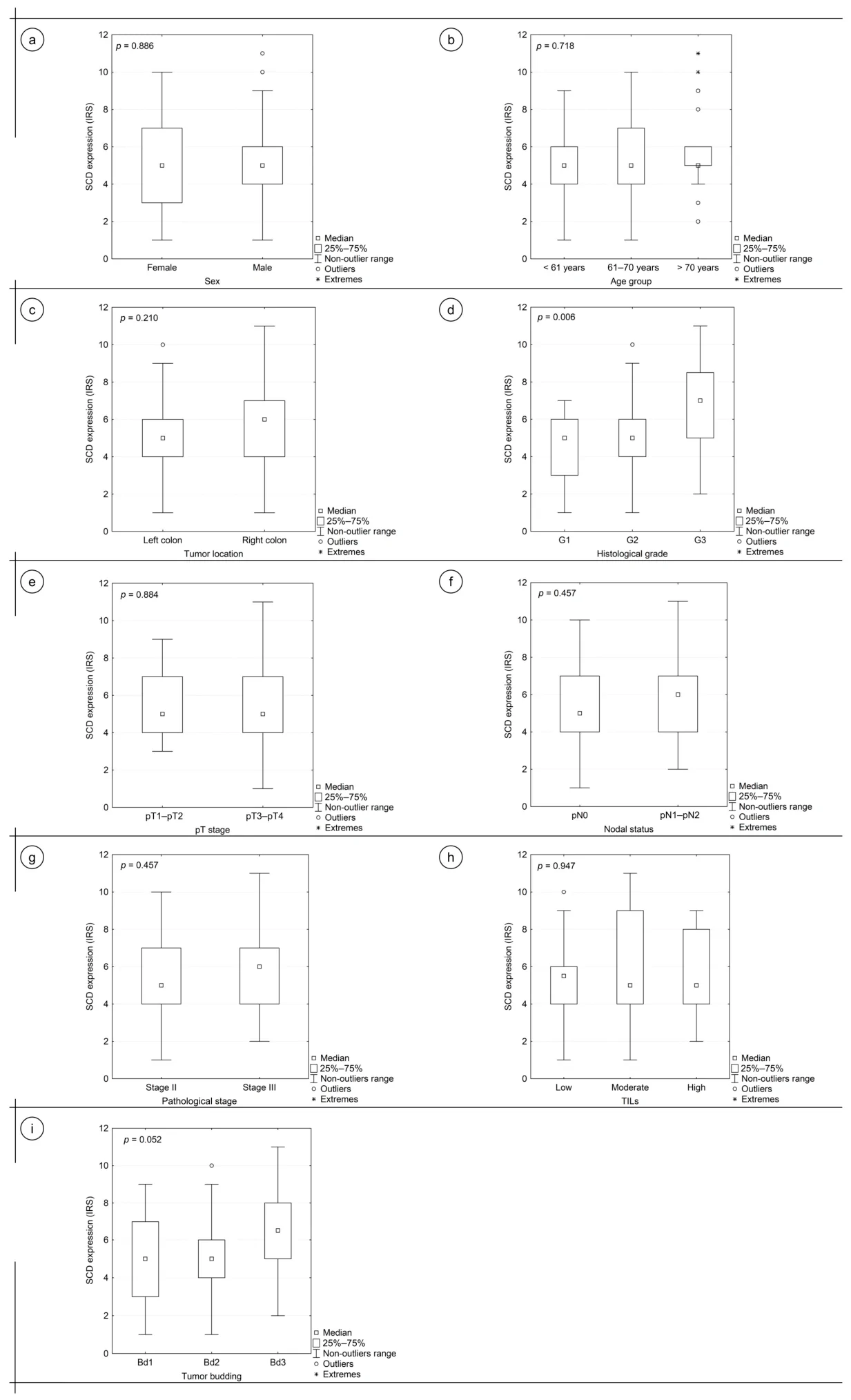
Association between SCD expression and clinicopathological parameters in colon adenocarcinoma. SCD expression was assessed by immunohistochemistry using the immunoreactive score (IRS). Boxplots show SCD expression according to sex (**a**), age group (**b**), tumour location (**c**), histological grade (**d**), pT stage (**e**), nodal status (**f**), pathological stage (**g**), tumour-infiltrating lymphocytes (TILs) (**h**), tumour budding (**i**). SCD expression did not differ significantly according to sex, age group, tumour location, pT stage, nodal status, pathological stage, TIL density. A significant association was observed with histological grade, with higher SCD expression in G3 tumours than in G1 and G2 tumours. Tumour budding showed a borderline association with SCD expression. Horizontal lines indicate medians, boxes indicate the 25th–75th percentiles, whiskers indicate the non-outlier range, circles indicate outliers, and asterisks indicate extreme values.

SCD IRS did not differ significantly according to sex, age group, tumour location, pT stage, nodal status, pathological stage, or tumour-infiltrating lymphocyte density (Figure 5; Table 3).

The strongest association was observed for histological grade. Mean SCD IRS values were 4.45 ± 1.86 in G1 tumours, 5.17 ± 2.18 in G2 tumours, and 6.83 ± 2.33 in G3 tumours. The difference across grades was statistically significant (*p* = 0.006; Figure 5d; Table 3), and post hoc analysis confirmed significantly higher SCD expression in G3 tumours compared with both G1 and G2 tumours. A positive correlation was also observed between increasing histological grade and SCD IRS values (ρ = 0.293, *p* = 0.002; Table 3). In Dunn post hoc comparisons with Holm adjustment, G3 tumours remained significantly higher than G1 tumours (adjusted *p* = 0.020) and G2 tumours (adjusted *p* = 0.011), whereas G1 and G2 tumours did not differ significantly (adjusted *p* = 0.424).

Tumour budding showed a borderline association with SCD expression. Bd3 tumours had the highest mean SCD IRS values, whereas Bd1 and Bd2 tumours showed lower and comparable values. However, this comparison did not reach conventional statistical significance (*p* = 0.052; Figure 5i; Table 3). Given the number of exploratory comparisons and the small subgroup sizes, this nominal borderline result was not interpreted as evidence of an association.

### 3.6. SCD Expression Is Positively Correlated with PCNA Expression

SCD IRS correlated positively with PCNA IRS (Spearman rho = 0.289, *p* = 0.002; Table 4). The modest magnitude of the association supported exploratory examination of their joint distribution but did not indicate a discrete biological class.

### 3.7. Secondary Exploratory Analysis of the Combined SCD–PCNA z-Score

**Figure 6:**
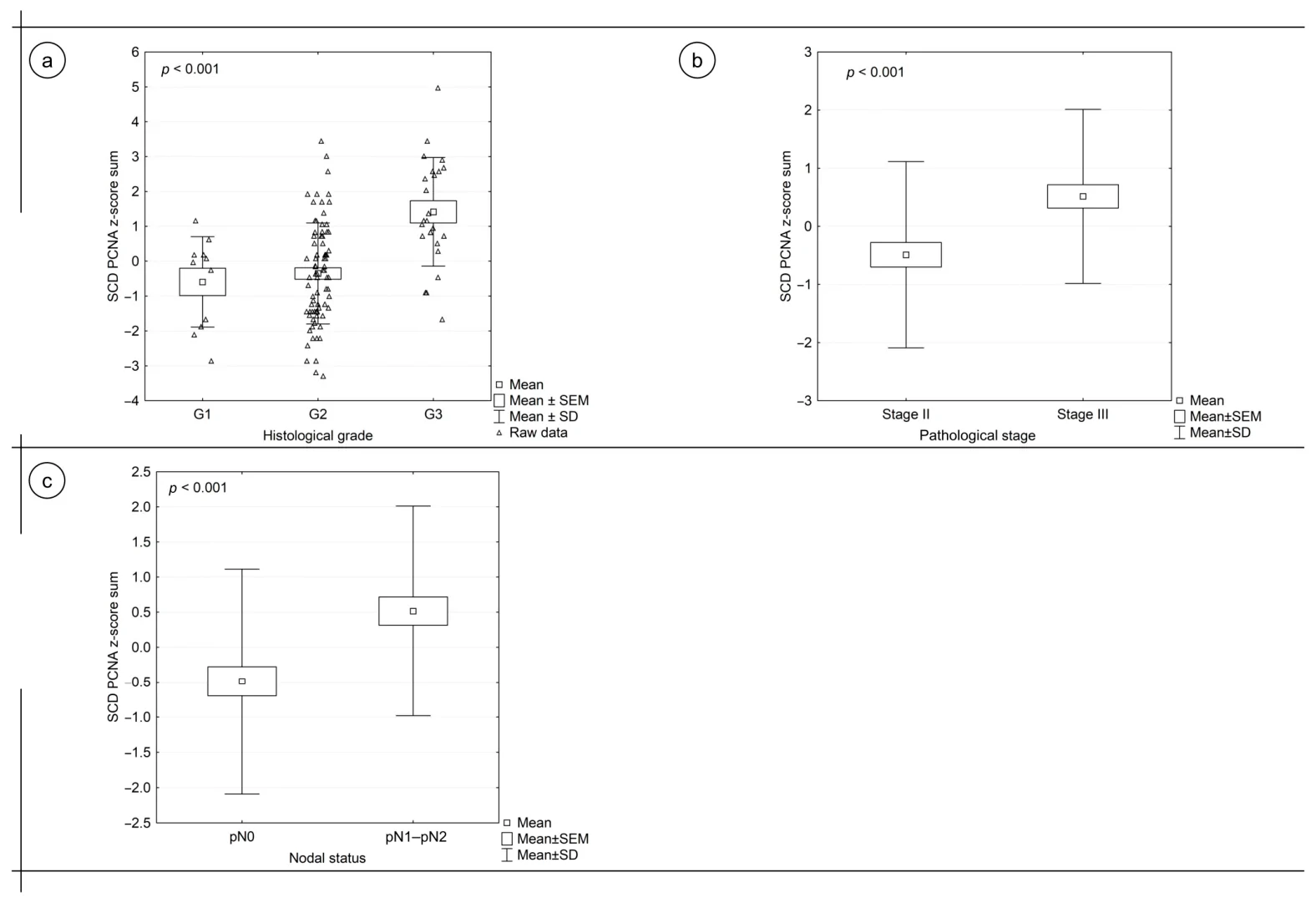
Secondary exploratory analysis of the combined SCD-PCNA z-score and selected clinicopathological features in colon adenocarcinoma. The combined score is a sample-dependent, equally weighted joint expression index and was analysed for hypothesis-generating purposes only. Panels show histological grade (**a**), pathological stage (**b**), and nodal status (**c**). The stage and nodal-status comparisons represent the same binary division of this cohort and should not be interpreted as independent findings. Squares indicate means, boxes indicate means ± SEM, whiskers indicate means ± SD, and triangles indicate individual raw data points where shown.

The combined SCD–PCNA z-score was evaluated as a secondary, equally weighted, sample-dependent exploratory index. It increased with histological grade and was higher in node-positive tumours (Table 5; Figure 6). Because stage II versus III represented the same patient division as nodal status, it was not considered an independent finding.

A secondary logistic-regression sensitivity analysis of the combined score and G3 histology was retained for completeness but moved to Supplementary Material S2 (Supplementary Methods and Table S1), because it does not address whether SCD adds information beyond PCNA.

### 3.8. Incremental Value of SCD Beyond PCNA

Nested models were used to test whether SCD added information beyond PCNA (Table 6). For G3 histology, adding SCD modestly improved the overall model fit (likelihood-ratio *p* = 0.044), but the adjusted SCD coefficient was borderline (OR = 1.273, 95% CI: 0.999–1.622, *p* = 0.051), and the increase in AUC from 0.784 to 0.810 was not statistically significant (DeLong *p* = 0.249). Because no global multiplicity adjustment was applied across the full set of analyses, the likelihood-ratio *p* = 0.044 should be regarded as a nominal exploratory finding rather than as confirmatory evidence of incremental value.

For lymph node involvement, SCD was not independently associated with the endpoint and did not improve either model fit or discrimination beyond PCNA. Thus, for G3 histology, addition of SCD was associated with only a modest nominal improvement in model fit and no statistically significant improvement in discrimination, whereas no incremental contribution was demonstrated for nodal involvement in the prespecified linear model.

Assessment of linearity in the logit showed no evidence of departure from linearity for either SCD IRS or PCNA IRS in the G3 histology model (SCD interaction *p* = 0.948; PCNA interaction *p* = 0.917; joint *p* = 0.993). For lymph node involvement, however, the exploratory assessment suggested possible non-linearity of the SCD effect (SCD interaction *p* = 0.035; PCNA interaction *p* = 0.121; joint *p* = 0.020). In an exploratory model allowing both linear and non-linear SCD terms, addition of SCD to the PCNA-only model was borderline overall (likelihood-ratio *p* = 0.054) and therefore did not establish additional predictive value.

### 3.9. Exploratory Assessment of Marker Separation Between Established G3 and G1/G2 Tumours

ROC analysis was retained only as an exploratory description of separation between tumours with established G3 histology and G1/G2 tumours; it was not intended to assess diagnostic accuracy, reclassification, or clinical utility. SCD IRS, PCNA IRS, and the combined SCD–PCNA z-score had AUCs of 0.705, 0.784, and 0.804, respectively (Table 7), and the combined score did not significantly outperform PCNA alone (DeLong *p* = 0.550). Detailed exploratory cut-offs, sensitivity, and specificity are provided in Supplementary Material S2, Table S2.

### 3.10. Exploratory ROC Analysis of Statistical Separation According to Lymph Node Involvement

For lymph node involvement, SCD IRS showed no meaningful discriminatory performance (AUC = 0.540), whereas PCNA IRS performed better (AUC = 0.751; Table 8). The combined SCD–PCNA z-score had a lower AUC than PCNA alone (0.676 versus 0.751), and a direct comparison favoured PCNA (*p* = 0.026). Thus, the composite score provided no improvement over PCNA for nodal involvement and was interpreted only as a hypothesis-generating joint expression index.

A secondary exploratory analysis restricted to G2 tumours is provided in Supplementary Material S2 (Supplementary Table S3 and Figure S2).

## 4. Discussion

In the present study, SCD expression in colon adenocarcinoma was evaluated primarily by immunohistochemistry in our institutional cohort, with supportive ultrastructural localisation. Public TCGA/UALCAN and CPTAC/UALCAN data were used only as supportive observations at the transcriptomic and proteomic levels. Consistent with previous reports, the tumour-associated increase in SCD observed in the present cohort should be regarded as confirmatory rather than novel; the principal additional contribution is the association with histological grade, together with its relationship with PCNA and supportive ultrastructural localisation. SCD expression was significantly higher in tumour tissue than in non-neoplastic mucosa and was higher in poorly differentiated G3 tumours, but it was not associated with nodal status or pathological stage. Analyses involving PCNA and the combined SCD–PCNA score were secondary and exploratory and were intended to assess biological context and incremental information.

The TCGA/UALCAN and CPTAC/UALCAN results should be regarded as supportive public-database observations. They are consistent with higher SCD expression in tumour than normal tissue at the transcriptomic and proteomic levels but are limited to platform-derived aggregate subgroup comparisons. No patient-level modelling, multivariable analysis, survival analysis, or independent replication of the institutional clinicopathological findings was performed. These public-database observations therefore do not constitute external validation of the institutional cohort; their role in the present study is limited to showing that tumour-associated SCD upregulation is also evident in transcriptomic and proteomic data sources.

The immunohistochemical analysis confirmed markedly higher SCD expression in tumour tissue than in corresponding non-neoplastic mucosa in 113 patient-matched pairs. This finding is consistent with previous studies showing that malignant cells may depend on SCD-mediated production of monounsaturated fatty acids to maintain lipid homeostasis and avoid saturated fatty acid-induced lipotoxic stress [6,9,11,12,13,23,24,25]. Experimental studies have also shown that inhibition of SCD1 can impair tumourigenic properties, promote tumour-cell apoptosis, and alter ceramide metabolism in colorectal cancer models [11,12,13]. Although the present study was not designed to establish causality, the strong tumour-associated increase in SCD supports its role as part of the metabolic adaptation of colon adenocarcinoma cells. Within the institutional cohort, the strongest clinicopathological association was observed with histological grade. G3 tumours showed significantly higher SCD IRS values than both G1 and G2 tumours, whereas no significant associations were found with sex, age, tumour location, pT stage, nodal status, pathological stage, and tumour-infiltrating lymphocyte density. These findings suggest that SCD expression may be more closely related to tumour dedifferentiation than to anatomical extent. Poorly differentiated tumours may require greater lipid remodelling and desaturation to support rapid growth, stress tolerance, and phenotypic plasticity, which is consistent with previous reports linking altered lipid metabolism with aggressive and stem-like tumour phenotypes [4,6,26,27,28]. Tumour budding showed only a borderline association with SCD expression. Bd3 tumours had the highest SCD IRS values, but the difference did not reach conventional statistical significance. This trend may indicate a possible relationship between lipid-metabolic activation and invasive morphology, particularly because tumour budding is associated with partial epithelial–mesenchymal transition and adverse behaviour in colorectal cancer [19,29]. However, this result should be regarded as preliminary and requires confirmation in larger cohorts.

These findings should be considered in the context of the existing colorectal cancer literature. Ran et al. showed that SCD1 promotes colorectal cancer migration and invasion in response to glucose through MUFA production and suppression of PTEN, with consequent modulation of downstream signalling and epithelial–mesenchymal transition [14]. Wu et al. linked the Nodal-SCD1 axis to colorectal cancer survival and metastasis through SCD1-mediated ferroptosis resistance [15]. Wang et al. subsequently integrated transcriptomic, immunohistochemical, single-cell, and functional data and further supported increased SCD expression and clinicopathological relevance in colorectal cancer [16]. The present results are therefore confirmatory with respect to tumour-associated SCD upregulation. In our institutional cohort, however, SCD expression was not significantly associated with nodal involvement or pathological stage; its strongest clinicopathological association was with histological grade, with the highest expression observed in G3 tumours. Differences from studies emphasising metastatic behaviour may reflect differences in cohort composition, endpoints, analytical approaches, and underlying biological heterogeneity. Accordingly, the present findings support the interpretation of SCD primarily as a correlate of tumour metabolic phenotype and histological dedifferentiation in this cohort rather than as a validated marker of metastatic potential.

SCD expression showed a modest positive correlation with PCNA, providing a proliferative context for the metabolic findings. The combined SCD–PCNA z-score was therefore retained only as a secondary exploratory index. Adding SCD to PCNA provided at most a small incremental contribution for G3 histology and did not significantly improve discrimination, whereas no incremental value was demonstrated for nodal involvement. Because histological grade and nodal status were already established by routine pathological examination, the ROC analyses should be interpreted only as descriptive assessments of separation between predefined groups rather than as evidence of diagnostic utility. The exploratory G2 subgroup analysis, reported in Supplementary Material S2, was likewise hypothesis-generating and should not be interpreted as a validated stratification approach. The absence of incremental value for nodal involvement refers to the prespecified linear model; an exploratory assessment suggested possible non-linearity of the SCD effect, but this finding requires confirmation in an independent cohort and was not considered evidence of clinical utility.

The immunogold TEM findings should likewise be interpreted as supportive. SCD association with endoplasmic reticulum-related membranes has been established previously, and the present ultrastructural observations were not intended to demonstrate a novel localisation pattern or provide mechanistic evidence. Because the TEM component was qualitative, no quantitative assessment of gold-particle density across subcellular compartments or patient groups was performed. Its contribution therefore lies in providing ultrastructural support for SCD localisation within the same tissue-based study rather than in establishing a previously unknown subcellular localisation pattern.

This study has several limitations. The institutional cohort was retrospective, single-centre, and restricted to pathological stages II and III because stage I and IV cases were only sparsely represented in the available archival material. This restriction limits the generalisability of the findings across the full spectrum of colon adenocarcinoma and may also have influenced the observed relationships between clinicopathological variables. Therefore, associations among pT category, nodal status, histological grade, tumour budding, and other pathological features should be interpreted within the context of this selected stage II–III cohort.

In addition, histological grade, tumour-infiltrating lymphocyte density, tumour budding were derived from the original archival histopathology reports. Consequently, the detailed assessment criteria applied to these variables could not be independently verified retrospectively unless explicitly documented in the original pathology records. The specific tumour-budding assessment protocol used in individual cases could likewise not be uniformly reconstructed from the available retrospective documentation. Associations involving these retrospectively recorded histopathological variables should therefore be interpreted with appropriate caution.

Importantly, future studies including the full spectrum of pathological stages and independent patient cohorts are required to determine whether the observed associations are generalisable beyond stage II–III disease.

The combined SCD–PCNA z-score was sample-dependent and equally weighted; no external validation was available, and no functional experiments were performed. Given the limited number of G3 events, the analysis used a multivariable logistic regression model with a limited number of prespecified covariates and should be interpreted as a limited robustness assessment rather than as a definitive predictive model. The TCGA/UALCAN and CPTAC/UALCAN components were limited to supportive, platform-based aggregate comparisons and did not include patient-level modelling, multivariable analysis, survival analysis, or independent replication of the institutional findings.

The lack of a direct comparison between PCNA and Ki-67 should also be acknowledged as a limitation, particularly because PCNA was used in several secondary analyses. Because PCNA was evaluated using an intensity-by-proportion IRS rather than a conventional labelling index, the resulting values should be interpreted as semi-quantitative measures of PCNA immunoreactivity rather than as direct estimates of the proliferative fraction.

The immunogold TEM component was qualitative and descriptive and was based on archival tissue material from 10 patients. No formal gold-particle counting or particle-density analysis was performed. Antibody-related control assessment was limited to a negative control with omission of the primary antibody, and no additional orthogonal antibody-validation procedures or repeat-staining reproducibility experiments were performed. The ultrastructural findings should therefore be interpreted only as supportive observations and not as quantitative evidence of SCD abundance or as evidence of a novel subcellular localisation pattern.

No a priori sample-size calculation was performed, and the small number of G3 events limits the precision and stability of regression and ROC estimates. The exploratory diagnostics suggested possible non-linearity of the SCD effect for nodal involvement, indicating that the prespecified linear model may provide an incomplete representation of this relationship.

An additional limitation concerns the reproducibility of the immunohistochemical assessment. Although slides were independently evaluated by two blinded observers and discrepant assessments were resolved by joint re-evaluation and consensus, observer-specific IRS values were not retained, and formal quantitative interobserver agreement could therefore not be calculated retrospectively. In addition, although negative controls were included, formal repeat-staining or technical replicate experiments were not performed, and staining reproducibility was therefore not quantitatively assessed.

A further limitation is the lack of molecular and metabolic covariates that may influence SCD expression and its association with histological grade. Mismatch-repair/MSI status, BRAF mutation status, and molecular subtype were not available for systematic analysis in the present retrospective cohort. These characteristics may be associated with tumour location, differentiation, immune infiltration, and metabolic phenotype and could therefore represent potential biological confounders. Detailed data on obesity, diabetes, and other metabolic comorbidities were likewise unavailable for systematic evaluation. Consequently, the observed association between SCD expression and histological grade should be interpreted as an unadjusted clinicopathological association rather than as evidence of an independent relationship after accounting for molecular or metabolic factors.

In addition, the number of subgroup comparisons, ROC analyses, regression models, nested-model comparisons, and secondary analyses was relatively large in relation to the overall cohort size. This increases the risk of unstable estimates and chance-positive findings, particularly in the small G1 (*n* = 11) and G3 (*n* = 24) subgroups. No formal correction for multiple testing was applied; therefore, *p* values from secondary and subgroup analyses should be considered nominal and hypothesis-generating. These findings require confirmation in larger independent cohorts, including the full spectrum of pathological stages before they can be regarded as robust.

The main contribution of the present study is not the demonstration of tumour-associated SCD upregulation itself, which has been reported previously, but the more detailed clinicopathological characterisation of SCD expression in the institutional cohort, particularly its association with histological grade. Its relationship with PCNA provides secondary proliferative context, while the immunogold TEM analysis provides supportive ultrastructural localisation. SCD expression itself was not associated with nodal involvement or pathological stage. Analyses involving PCNA were secondary and exploratory: adding SCD to PCNA yielded only a modest nominal improvement in model fit for G3 histology, no statistically significant improvement in discrimination, and no incremental value for nodal involvement. The combined SCD–PCNA score should therefore remain a hypothesis-generating analytical construct rather than be interpreted as a new biological classification or a clinically superior biomarker. Independent validation, outcome-based analyses, and functional studies are required before broader biological or clinical interpretation.

## 5. Conclusions

Consistent with previous reports, SCD expression was increased in colon adenocarcinoma compared with non-neoplastic mucosa. The principal contribution of the present study is the observation that within the institutional cohort, SCD expression was associated primarily with histological dedifferentiation, together with a modest relationship with PCNA expression and supportive ultrastructural localisation. These findings extend existing evidence on SCD biology in colorectal cancer rather than establishing tumour-associated SCD upregulation as a novel observation. However, SCD expression alone was not associated with nodal involvement, and the addition of SCD to PCNA did not significantly improve discriminatory performance. Therefore, the present findings do not support the use of either SCD alone or the combined SCD–PCNA score in routine pathological assessment or clinical decision-making. Their potential clinical relevance requires validation in independent cohorts using clinically relevant patient outcomes and formal assessment of incremental value beyond established clinicopathological parameters.

## Figures and Tables

**Table 1:** Clinicopathological characteristics of the study cohort.

Characteristic	Category	*n* (%) or Value
Demographic characteristics		
Total patients		113
Sex	Female	49 (43.4)
	Male	64 (56.6)
Age, years	Mean ± SD	66.6 ± 9.2
	Median [Q1–Q3]	66 [63–75]
	Range	45–90
Age group	≤60 years	29 (25.7)
	61–70 years	47 (41.6)
	>70 years	37 (32.7)
Tumour characteristics		
Tumour location	Left colon	66 (58.4)
	Right colon	47 (41.6)
Histological grade	G1	11 (9.7)
	G2	78 (69.0)
	G3	24 (21.2)
pT stage	pT1	3 (2.7)
	pT2	11 (9.7)
	pT3	82 (72.6)
	pT4	17 (15.0)
pN stage	N0	58 (51.3)
	N1	41 (36.3)
	N2	14 (12.4)
Pathological stage	Stage I	0 (0.0)
	Stage II	58 (51.3)
	Stage III	55 (48.7)
Histopathological and microenvironmental features		
Tumour-infiltrating lymphocytes	Low	60 (53.1)
	Moderate	38 (33.6)
	High	15 (13.3)
Tumour budding	Bd1	48 (42.5)
	Bd2	45 (39.8)
	Bd3	20 (17.7)

**Abbreviations:** SD, standard deviation; Q1–Q3, first–third quartile; pT, pathological tumour stage; pN, pathological nodal stage; TILs, tumour-infiltrating lymphocytes; Bd, tumour budding grade.

**Table 2:** Paired comparison of SCD expression between colon adenocarcinoma tissue and corresponding non-neoplastic colonic mucosa (*n* = 113 patient-matched pairs).

Tissue Type	Mean ± SD	Median [Q1–Q3]	Min–Max	Statistical Analysis
Colon adenocarcinoma tissue	5.45 ± 2.29	5 [4–7]	1–11	Wilcoxon signed-rank test: W = 0; Z = −9.006; *p* < 0.001
Non-neoplastic colonic mucosa	1.79 ± 0.95	2 [1–2]	1–5	

Abbreviations: SCD, stearoyl-CoA desaturase; IRS, immunoreactive score; SD, standard deviation; Q1–Q3, first–third quartile; W, Wilcoxon signed-rank statistic; Z, standardised Wilcoxon test statistic.

**Table 3:** Association between SCD expression and clinicopathological characteristics in colon adenocarcinoma.

Variable	Category	*n*	Mean ± SD	Median [Q1–Q3]	Min–Max	Statistical Analysis
Overall cohort		113	5.45 ± 2.29	5 [4–7]	1–11	–
Demographic characteristics						
Sex	Female	49	5.39 ± 2.56	5 [3–7]	1–10	Z = −0.144; *p* = 0.886
	Male	64	5.50 ± 2.09	5 [4–6]	1–11	
Age group	≤60 years	29	5.21 ± 2.21	5 [4–6]	1–9	H = 0.662; *p* = 0.718 ρ = 0.092; *p* = 0.332
	61–70 years	47	5.34 ± 2.43	5 [4–7]	1–10	
	>70 years	37	5.78 ± 2.20	5 [5–6]	2–11	
Tumour characteristics						
Tumour location	Left colon	66	5.24 ± 2.16	5 [4–6]	1–10	Z = −1.255; *p* = 0.210
	Right colon	47	5.74 ± 2.45	6 [4–7]	1–11	
Histological grade	G1	11	4.45 ± 1.86	5 [3–6]	1–7	H = 10.261; *p* = 0.006 Post hoc: G1 < G3 *, G2 < G3 * ρ = 0.293; *p* = 0.002
	G2	78	5.17 ± 2.18	5 [4–6]	1–10	
	G3	24	6.83 ± 2.33	7 [5–8.5]	2–11	
pT stage	pT1/pT2	14	5.64 ± 2.13	5 [4–7]	3–9	Z = 0.145; *p* = 0.884 ρ = 0.028; *p* = 0.771
	pT3/pT4	99	5.42 ± 2.32	5 [4–7]	1–11	
pN stage	N0	58	5.29 ± 2.44	5 [4–7]	1–10	Z = −0.743; *p* = 0.457 ρ = 0.074; *p* = 0.439
	N1/N2	55	5.62 ± 2.14	6 [4–7]	2–11	
Pathological stage	Stage II	58	5.29 ± 2.44	5 [4–7]	1–10	Z = −0.743; *p* = 0.457
	Stage III	55	5.62 ± 2.14	6 [4–7]	2–11	
Histopathological and microenvironmental features						
Tumour-infiltrating lymphocytes	Low	60	5.30 ± 1.94	5.5 [4–6]	1–10	H = 0.108; *p* = 0.947 ρ = 0.018; *p* = 0.846
	Moderate	38	5.68 ± 2.75	5 [4–9]	1–11	
	High	15	5.47 ± 2.42	5 [4–8]	2–9	
Tumour budding	Bd1	48	5.17 ± 2.42	5 [3–7]	1–9	H = 5.921; *p* = 0.052 ρ = 0.172; *p* = 0.068
	Bd2	45	5.24 ± 2.06	5 [4–6]	1–10	
	Bd3	20	6.60 ± 2.23	6.5 [5–8]	2–11	

Abbreviations: SCD, stearoyl-CoA desaturase; IRS, immunoreactive score; SD, standard deviation; Q1–Q3, first–third quartile; pT, pathological tumour stage; pN, pathological nodal stage; TILs, tumour-infiltrating lymphocytes; Bd, tumour budding grade; Z, Mann–Whitney U test statistic; H, Kruskal–Wallis test statistic; ρ, Spearman correlation coefficient. * Post hoc comparison significant at *p* < 0.05. Post hoc comparisons were performed using Dunn’s test with Holm adjustment.

**Table 4:** Correlation between SCD IRS and PCNA IRS expression in colorectal cancer.

Variables	*n*	Spearman’s Rho	*p* Value
SCD IRS cancer vs. PCNA IRS cancer	113	0.289	0.002

Abbreviations: SCD, stearoyl-CoA desaturase; PCNA, proliferating cell nuclear antigen; IRS, immunoreactive score; rho, Spearman correlation coefficient.

**Table 5:** Association between the combined SCD-PCNA z-score and selected clinicopathological characteristics in colon adenocarcinoma.

Variable	Category	*n*	Mean ± SD	Median [Q1–Q3]	Min–Max	Statistical Analysis
Overall cohort						
Overall cohort		113	0.00 ± 1.63	0.08 [−1.33–1.06]	−3.29–4.97	–
Histological differentiation						
Histological grade	G1	11	−0.59 ± 1.30	−0.02 [−1.88–0.19]	−2.85–1.17	F = 14.471; *p* < 0.001 Post hoc: G1 < G3 ***; G2 < G3 ***
	G2	78	−0.35 ± 1.45	−0.41 [−1.44–0.73]	−3.29–3.45	
	G3	24	1.42 ± 1.56	1.17 [0.62–2.58]	−1.66–4.97	
Histological grade, grouped	G1/G2	89	−0.38 ± 1.43	−0.36 [−1.44–0.62]	−3.29–3.45	t = −5.373; *p* < 0.001 ρ = 0.396; *p* < 0.001
	G3	24	1.42 ± 1.56	1.17 [0.62–2.58]	−1.66–4.97	
Stage and nodal involvement						
Pathological stage	Stage II	58	−0.49 ± 1.60	−0.90 [−1.56–0.73]	−3.29–3.45	t = −3.438; *p* < 0.001
	Stage III	55	0.52 ± 1.50	0.52 [−0.46–1.17]	−2.21–4.97	
pN stage	N0	58	−0.49 ± 1.60	−0.90 [−1.56–0.73]	−3.29–3.45	t = −3.438; *p* < 0.001
	N1/N2	55	0.52 ± 1.50	0.52 [−0.46–1.17]	−2.21–4.97	

**Abbreviations:** SCD, stearoyl-CoA desaturase; PCNA, proliferating cell nuclear antigen; SD, standard deviation; Q1–Q3, first–third quartile; pN, pathological nodal stage; F, one-way ANOVA statistic; t, Student’s *t*-test statistic; ρ, Spearman correlation coefficient. *** *p* < 0.001 in post hoc comparisons. Pairwise comparisons following ANOVA were performed using Tukey’s HSD test.

**Table 6:** Incremental value of SCD beyond PCNA in nested logistic regression models.

Endpoint and Metric	Model A: PCNA	Model B: PCNA + SCD	Increment or Comparison	Interpretation
G3 histology: AIC/pseudo-R^2^	98.68/0.190	96.63/0.224	ΔAIC = −2.05; ΔR^2^ = +0.034	Modest improvement in model fit
G3 histology: AUC (95% CI)	0.784 (0.681–0.886)	0.810 (0.709–0.910)	ΔAUC = +0.026	No significant improvement in discrimination
G3 histology: model comparisons	—	LR χ^2^ = 4.044	LRT *p* = 0.044; DeLong *p* = 0.249	Model fit improved modestly; AUC did not
Nodal involvement: AIC/pseudo-R^2^	137.65/0.146	139.06/0.150	ΔAIC = +1.41; ΔR^2^ = +0.004	No meaningful improvement in model fit
Nodal involvement: AUC (95% CI)	0.751 (0.663–0.839)	0.757 (0.668–0.845)	ΔAUC = +0.006	No significant improvement in discrimination
Nodal involvement: model comparisons	—	LR χ^2^ = 0.591	LRT *p* = 0.442; DeLong *p* = 0.497	No evidence of incremental value

**Abbreviations:** AIC, Akaike information criterion; AUC, area under the receiver operating characteristic curve; CI, confidence interval; LRT, likelihood-ratio test; PCNA, proliferating cell nuclear antigen; SCD, stearoyl-CoA desaturase. Δ values were calculated as Model B minus Model A.

**Table 7:** Exploratory ROC analysis of statistical separation between tumours with established G3 histology and G1/G2 colon adenocarcinomas.

Marker	AUC	95% CI	*p* Value
SCD IRS	0.705	0.585–0.824	0.002
PCNA IRS	0.784	0.681–0.886	<0.001
Combined SCD-PCNA z-score	0.804	0.703–0.904	<0.001

**Abbreviations:** ROC, receiver operating characteristic; AUC, area under the curve; CI, confidence interval; SCD, stearoyl-CoA desaturase; PCNA, proliferating cell nuclear antigen; IRS, immunoreactive score. Detailed Youden-derived cut-offs, sensitivity, and specificity are reported in Supplementary Material S2, Table S2. The reported *p* values test the null hypothesis that AUC = 0.5. Pairwise comparisons between ROC curves were performed using the DeLong test and are reported separately in the text.

**Table 8:** Exploratory ROC analysis of statistical separation according to lymph node involvement (pN1/pN2) in colon adenocarcinoma.

Marker	AUC	95% CI	*p* Value
SCD IRS	0.540	0.434–0.647	0.460
PCNA IRS	0.751	0.663–0.839	<0.001
Combined SCD-PCNA z-score	0.676	0.576–0.776	0.001

**Abbreviations:** ROC, receiver operating characteristic; AUC, area under the curve; CI, confidence interval; SCD, stearoyl-CoA desaturase; PCNA, proliferating cell nuclear antigen; IRS, immunoreactive score; pN, pathological nodal stage. The reported ROC estimates are apparent, exploratory same-cohort estimates and were not internally or independently validated. Cut-off, sensitivity, and specificity estimates were omitted from the main table to avoid overemphasising threshold-based performance. The reported *p* values test the null hypothesis that AUC = 0.5. Pairwise comparisons between ROC curves were performed using the DeLong test and are reported separately in the text.

## Data Availability

The datasets generated and analysed during the current study are available from the corresponding author upon reasonable request.

## Supplementary Materials

The following supporting information can be downloaded at: https://www.mdpi.com/article/10.3390/jcm15176817/s1. Supplementary Material S1: Figure S1. Negative controls for SCD immunohistochemical staining in non-neoplastic colonic mucosa and colon adenocarcinoma. Supplementary Material S2: Table S1. Logistic regression analysis of the association between the combined SCD-PCNA z-score and poorly differentiated G3 colon adenocarcinoma. Table S2. Apparent exploratory ROC threshold estimates derived in the same institutional cohort (Panels A and B). Table S3. Subgroup analysis of the combined SCD-PCNA z-score in moderately differentiated G2 colon adenocarcinomas. Figure S2. Exploratory subgroup analysis of the combined SCD-PCNA z-score in moderately differentiated G2 colon adenocarcinomas. Supplementary Material S3: SCD database.
