# Supplementary material for: Clinicopathological Significance of SCD Expression in Colon Adenocarcinoma: Association with Histological Grade and Exploratory Assessment in Relation to PCNA
Source: J Clin Med. 2026 Sep 2;15(17):6817. doi: 10.3390/jcm15176817 (PMC13566249; doi:10.3390/jcm15176817)
Supplement: Supplementary file 1 [file jcm-15-06817-s001.zip › Supplementary Material S3.pdf]

	Sex	Age	Diagnosis	mor Locati	Grade	pT	pN	Stage
	M	67	Colon aden	Left colon	G2	pT3	pN0	II
	F	66	Colon aden	Right colon	G2	pT4	pN0	II
	M	73	Colon aden	Right colon	G2	pT3	pN0	II
	M	83	Colon aden	Left colon	G2	pT4	pN0	II
	M	81	Colon aden	Right colon	G2	pT3	pN1	III
	F	72	Colon aden	Left colon	G2	pT3	pN0	II
	M	74	Colon aden	Left colon	G2	pT3	pN0	II
	M	68	Colon aden	Left colon	G2	pT3	pN2	III
	M	72	Colon aden	Left colon	G2	pT3	pN0	II
	F	86	Colon aden	Right colon	G2	pT1	pN2	III
	F	70	Colon aden	Left colon	G2	pT3	pN0	II
	F	63	Colon aden	Left colon	G2	pT3	pN0	II
	M	50	Colon aden	Right colon	G1	pT2	pN1	III
	F	72	Colon aden	Left colon	G2	pT3	pN1	III
	F	90	Colon aden	Left colon	G2	pT3	pN1	III
	F	56	Colon aden	Left colon	G2	pT3	pN1	III
	F	62	Colon aden	Right colon	G1	pT3	pN0	II
	F	68	Colon aden	Left colon	G2	pT3	pN1	III
	M	64	Colon aden	Right colon	G3	pT3	pN2	III
	M	63	Colon aden	Left colon	G1	pT4	pN0	II
	F	74	Colon aden	Left colon	G1	pT3	pN0	II
	M	67	Colon aden	Right colon	G2	pT4	pN0	II
	M	79	Colon aden	Right colon	G2	pT3	pN1	III
	M	61	Colon aden	Right colon	G2	pT3	pN1	III
	F	77	Colon aden	Right colon	G2	pT3	pN0	II
	F	60	Colon aden	Right colon	G2	pT3	pN0	II
	M	67	Colon aden	Right colon	G2	pT2	pN1	III
	M	67	Colon aden	Left colon	G2	pT3	pN0	II
	M	63	Colon aden	Left colon	G2	pT3	pN1	III
	M	77	Colon aden	Left colon	G2	pT3	pN0	II
	F	66	Colon aden	Right colon	G3	pT4	pN0	II
	F	67	Colon aden	Left colon	G1	pT3	pN0	II
	M	65	Colon aden	Left colon	G2	pT3	pN2	III
	F	63	Colon aden	Right colon	G3	pT3	pN0	II
	M	52	Colon aden	Right colon	G2	pT3	pN1	III
	F	62	Colon aden	Right colon	G2	pT3	pN0	II
	M	55	Colon aden	Left colon	G2	pT3	pN0	II
	M	55	Colon aden	Left colon	G2	pT3	pN0	II
	M	77	Colon aden	Left colon	G2	pT3	pN1	III
	F	61	Colon aden	Left colon	G3	pT3	pN0	II
	M	90	Colon aden	Left colon	G2	pT2	pN1	III
	M	73	Colon aden	Right colon	G3	pT3	pN2	III
	M	70	Colon aden	Right colon	G2	pT3	pN1	III
	F	61	Colon aden	Left colon	G2	pT3	pN0	II
	F	67	Colon aden	Left colon	G2	pT3	pN0	II
	M	63	Colon aden	Left colon	G2	pT2	pN1	III

M	73	Colon aden	Left colon	G1	pT3	pN0	II
M	69	Colon aden	Right colon	G2	pT4	pN0	II
F	79	Colon aden	Left colon	G1	pT4	pN0	II
F	63	Colon aden	Left colon	G2	pT2	pN1	III
F	66	Colon aden	Right colon	G2	pT3	pN0	II
F	83	Colon aden	Left colon	G2	pT3	pN1	III
M	79	Colon aden	Right colon	G3	pT3	pN0	II
F	68	Colon aden	Left colon	G2	pT4	pN0	II
F	66	Colon aden	Left colon	G2	pT3	pN0	II
M	77	Colon aden	Right colon	G2	pT3	pN1	III
M	58	Colon aden	Left colon	G1	pT3	pN2	III
M	78	Colon aden	Left colon	G2	pT3	pN0	II
F	75	Colon aden	Left colon	G3	pT3	pN1	III
M	58	Colon aden	Right colon	G2	pT3	pN1	III
F	64	Colon aden	Right colon	G3	pT3	pN0	II
F	72	Colon aden	Right colon	G2	pT2	pN2	III
F	77	Colon aden	Left colon	G3	pT3	pN1	III
M	59	Colon aden	Left colon	G2	pT4	pN0	II
M	69	Colon aden	Left colon	G2	pT3	pN2	III
F	60	Colon aden	Left colon	G2	pT3	pN0	II
M	73	Colon aden	Left colon	G2	pT4	pN1	III
M	63	Colon aden	Left colon	G3	pT3	pN2	III
F	70	Colon aden	Left colon	G3	pT3	pN0	II
M	64	Colon aden	Left colon	G2	pT1	pN1	III
F	58	Colon aden	Left colon	G2	pT2	pN1	III
F	50	Colon aden	Left colon	G3	pT2	pN2	III
M	66	Colon aden	Left colon	G3	pT3	pN0	II
F	64	Colon aden	Left colon	G3	pT3	pN1	III
M	61	Colon aden	Left colon	G2	pT3	pN1	III
M	81	Colon aden	Right colon	G3	pT4	pN0	II
M	72	Colon aden	Left colon	G3	pT3	pN0	II
F	79	Colon aden	Right colon	G3	pT3	pN0	II
F	61	Colon aden	Right colon	G2	pT4	pN1	III
M	72	Colon aden	Left colon	G3	pT3	pN0	II
M	55	Colon aden	Right colon	G2	pT4	pN0	II
M	78	Colon aden	Right colon	G1	pT3	pN1	III
M	57	Colon aden	Right colon	G1	pT3	pN1	III
M	50	Colon aden	Left colon	G2	pT3	pN2	III
F	45	Colon aden	Right colon	G3	pT3	pN0	II
F	57	Colon aden	Right colon	G2	pT3	pN0	II
F	72	Colon aden	Right colon	G2	pT3	pN0	II
M	70	Colon aden	Right colon	G2	pT3	pN0	II
F	66	Colon aden	Left colon	G2	pT3	pN0	II
M	60	Colon aden	Right colon	G1	pT3	pN0	II
F	58	Colon aden	Left colon	G2	pT2	pN1	III
M	59	Colon aden	Right colon	G3	pT4	pN0	II
F	71	Colon aden	Left colon	G2	pT3	pN0	II
M	61	Colon aden	Left colon	G2	pT3	pN1	III

M	81	Colon aden	Left colon	G3	pT3	pN1	III
M	68	Colon aden	Right colon	G2	pT3	pN0	II
F	57	Colon aden	Left colon	G2	pT3	pN1	III
F	83	Colon aden	Left colon	G2	pT4	pN1	III
M	57	Colon aden	Left colon	G2	pT4	pN2	III
F	60	Colon aden	Right colon	G3	pT3	pN1	III
M	54	Colon aden	Right colon	G2	pT3	pN0	II
M	50	Colon aden	Right colon	G2	pT3	pN0	II
M	70	Colon aden	Left colon	G2	pT4	pN2	III
F	67	Colon aden	Right colon	G2	pT3	pN0	II
F	66	Colon aden	Right colon	G2	pT3	pN2	III
M	74	Colon aden	Left colon	G2	pT3	pN1	III
M	59	Colon aden	Right colon	G2	pT2	pN1	III
M	59	Colon aden	Left colon	G2	pT3	pN0	II
M	72	Colon aden	Left colon	G2	pT3	pN1	III
M	61	Colon aden	Right colon	G3	pT3	pN0	II
F	61	Colon aden	Left colon	G2	pT2	pN1	III
M	58	Colon aden	Right colon	G3	pT1	pN1	III
M	46	Colon aden	Left colon	G2	pT3	pN1	III

TILs	ımor Budding	SCD IRS canc	NA IRS can	SCD Healthy
Moderate	Bd3	8	5	3
Moderate	Bd3	9	8	3
Moderate	Bd2	5	5	2
Moderate	Bd3	9	8	2
Moderate	Bd2	3	6	2
High	Bd3	4	5	1
Low	Bd2	3	4	1
Low	Bd2	5	9	2
Low	Bd3	5	5	3
Low	Bd2	6	10	2
Moderate	Bd1	1	8	1
Low	Bd1	4	4	1
High	Bd1	4	8	1
Low	Bd1	3	9	1
High	Bd1	3	9	1
Low	Bd3	8	10	1
Moderate	Bd1	2	7	1
Moderate	Bd1	2	6	1
Moderate	Bd1	4	10	2
Moderate	Bd1	1	7	1
Moderate	Bd1	5	8	2
Low	Bd1	6	5	2
Moderate	Bd1	6	7	2
Moderate	Bd3	6	9	2
Moderate	Bd2	2	6	1
Low	Bd2	1	8	1
Low	Bd2	4	8	2
Low	Bd2	5	5	2
Low	Bd2	6	10	2
Moderate	Bd1	5	8	2
High	Bd2	8	7	2
Low	Bd1	3	4	1
Low	Bd1	3	7	1
Low	Bd1	7	8	1
Moderate	Bd2	5	9	1
High	Bd3	2	4	1
Moderate	Bd2	5	4	1
High	Bd1	9	6	3
Low	Bd2	6	6	3
Moderate	Bd3	10	8	3
Low	Bd2	5	8	2
Moderate	Bd3	11	12	1
Low	Bd2	10	10	1
Low	Bd2	6	4	1
Low	Bd2	5	5	1
Low	Bd3	7	7	2

Low	Bd2	5	8	2
Moderate	Bd1	9	5	1
High	Bd1	4	5	1
Low	Bd1	3	8	1
Low	Bd3	6	5	1
Low	Bd1	6	7	1
Low	Bd2	4	11	2
Moderate	Bd1	9	6	1
Moderate	Bd1	9	8	1
High	Bd1	9	6	1
Low	Bd2	6	7	2
Low	Bd1	9	7	2
High	Bd2	6	9	2
Low	Bd2	6	6	2
High	Bd1	8	10	2
Low	Bd1	9	10	1
Low	Bd3	8	10	1
Moderate	Bd1	5	7	1
Low	Bd2	4	8	1
Low	Bd2	5	5	1
Low	Bd2	6	9	1
Low	Bd3	5	9	2
Low	Bd1	7	7	2
Low	Bd1	4	10	2
Moderate	Bd2	5	8	2
Moderate	Bd1	9	9	3
Low	Bd2	5	9	3
Moderate	Bd2	4	9	1
Low	Bd1	4	7	1
Moderate	Bd2	5	6	1
Moderate	Bd1	5	6	2
Moderate	Bd2	10	10	3
High	Bd1	7	8	1
Low	Bd1	6	9	1
Moderate	Bd2	3	7	1
Low	Bd1	6	9	1
Low	Bd2	6	8	3
Low	Bd1	4	5	3
High	Bd2	7	11	1
Moderate	Bd3	4	6	1
Low	Bd1	6	6	1
Low	Bd1	6	7	1
Low	Bd1	1	5	1
Low	Bd3	7	6	4
Moderate	Bd2	6	6	4
High	Bd1	2	7	1
Low	Bd1	5	5	2
Low	Bd2	6	7	2

Low	Bd2	10	9	3
Low	Bd2	3	4	3
Moderate	Bd1	2	7	1
Moderate	Bd2	5	5	1
Low	Bd2	2	6	1
Moderate	Bd2	8	9	1
Low	Bd2	2	8	1
Moderate	Bd1	5	6	4
Low	Bd1	7	8	2
Low	Bd2	4	6	2
Low	Bd3	7	7	3
Low	Bd3	5	7	3
Moderate	Bd1	5	9	3
High	Bd3	5	8	2
High	Bd2	4	7	2
Low	Bd3	6	6	4
Low	Bd1	3	7	3
Moderate	Bd2	9	10	5
Low	Bd1	6	10	5
