# Supplementary material for: Clinicopathological Significance of SCD Expression in Colon Adenocarcinoma: Association with Histological Grade and Exploratory Assessment in Relation to PCNA
Source: J Clin Med. 2026 Sep 2;15(17):6817. doi: 10.3390/jcm15176817 (PMC13566249; doi:10.3390/jcm15176817)
Supplement: Supplementary file 1 [file jcm-15-06817-s001.zip › Supplementary Material S2.pdf]

Supplementary Material S2

Clinicopathological Significance of SCD Expression in Colon Adenocarcinoma: Association with Histological Grade and Exploratory Assessment in Relation to PCNA

Supplementary Methods: secondary logistic-regression sensitivity analysis

Logistic regression was used to examine the association between the combined SCD-PCNA z-score and poorly differentiated G3 histology. The univariable model included the combined z-score as the sole predictor, whereas the multivariable model was adjusted for age, sex, and tumour location. Results were reported as odds ratios (ORs) with 95% confidence intervals (CIs). Age, sex, and tumour location were selected a priori as a limited set of prespecified clinicodemographic covariates that could plausibly influence tumour biology or marker expression while remaining external to the histological endpoint. Because only 24 tumours were classified as G3, additional adjustment for multiple pathological characteristics was avoided to limit overfitting and unstable coefficient estimates; pT, pN, tumour budding and tumour-infiltrating lymphocyte density were considered parallel manifestations of tumour aggressiveness rather than established confounders of the marker-grade association. The adjusted model was therefore intended as a limited robustness analysis rather than a comprehensive model of independent predictors of G3 histology.

Supplementary Results: association of the combined SCD-PCNA z-score with G3 histology

In this secondary sensitivity analysis, increasing combined z-score values were associated with higher odds of G3 histology in the univariable model (OR = 2.253, 95% CI: 1.541-3.295, $p < 0.001$; Supplementary Table S1). The association remained significant after adjustment for age, sex, and tumour location (OR = 2.190, 95% CI: 1.499-3.199, $p < 0.001$). Age, sex, and tumour location were not independently associated with G3 status. This analysis is provided for completeness and should be interpreted as a robustness analysis of the composite-score association rather than evidence that the score provides incremental value beyond PCNA.

Supplementary Table S1. Logistic regression analysis of the association between the combined SCD-PCNA z-score and poorly differentiated G3 colon adenocarcinoma.

Variable	Model 1 OR (95% CI)	p value	Model 2 OR (95% CI)	p value
Combined SCD-PCNA z-score	2.253 (1.541-3.295)	< 0.001	2.190 (1.499-3.199)	< 0.001
Age	-	-	0.998 (0.945-1.054)	0.934
Sex	-	-	0.772 (0.254-2.054)	0.541
Tumour location	-	-	1.414 (0.497-4.028)	0.516

Model 1: univariable logistic regression model including the combined SCD-PCNA z-score only.

Model 2: multivariable logistic regression model adjusted for age, sex, and tumour location.

Abbreviations: OR, odds ratio; CI, confidence interval; SCD, stearoyl-CoA desaturase; PCNA, proliferating cell nuclear antigen.

Supplementary Table S2. Apparent exploratory ROC threshold estimates derived in the same institutional cohort.

Panel A presents grade-based thresholds for separation of tumours with established G3 histology from G1/G2 tumours. Panel B presents thresholds for lymph node involvement (pN1/pN2). All cut-offs, sensitivities, and specificities were derived and evaluated in the same 113-patient cohort and should therefore be regarded as apparent, exploratory estimates rather than validated diagnostic performance.

Panel A. Established G3 histology versus G1/G2 tumours.

Marker	AUC	95% CI	p value	Optimal cut-off	Sensitivity (%)	Specificity (%)
SCD IRS	0.705	0.585-0.824	0.002	7	54.17	80.90
PCNA IRS	0.784	0.681-0.886	< 0.001	9	66.67	83.15
Combined SCD-PCNA z-score	0.804	0.703-0.904	< 0.001	0.292	83.33	70.79

Abbreviations: ROC, receiver operating characteristic; AUC, area under the curve; CI, confidence interval; SCD, stearoyl-CoA desaturase; PCNA, proliferating cell nuclear antigen; IRS, immunoreactive score. Cut-off values were determined using the Youden index in the same study cohort and should be interpreted as exploratory and potentially optimistic rather than clinically applicable thresholds.

Panel B. Lymph node involvement (pN1/pN2).

Marker	AUC	95% CI	p value	Optimal cut-off	Sensitivity (%)	Specificity (%)
SCD IRS	0.540	0.434–0.647	0.460	–	–	–
PCNA IRS	0.751	0.663–0.839	< 0.001	7	83.64	53.45
Combined SCD-PCNA z-score	0.676	0.576–0.776	0.001	−0.792	85.45	53.45

For SCD IRS, no operating threshold is reported because the AUC was not statistically different from 0.5. Cut-offs were determined using the Youden index. These same-cohort operating characteristics were not internally or independently validated.

Supplementary Results. Exploratory subgroup analysis in G2 tumours

A secondary exploratory analysis of the combined SCD–PCNA z-score was performed in G2 tumours and was not intended to establish a clinical stratification approach.

Node-positive G2 tumours had higher combined scores than node-negative tumours (p = 0.001; Figure S1a; Table S3), while no differences were observed for tumour budding or tumour-infiltrating lymphocyte density. Because pathological stage II versus III was determined by nodal status in this cohort and SCD did not add information beyond PCNA, this observation remained hypothesis-generating.

Supplementary Table S3. Subgroup analysis of the combined SCD-PCNA z-score in moderately differentiated G2 colon adenocarcinomas.

**Variable**	**Category**	n	**Mean ± SD**	**Median [Q1–Q3]**	**Min–Max**	**Statistical analysis**
Nodal and stage-related features						
pN stage	N0	37	−0.90 ± 1.39	−1.33 [−1.56–0.08]	−3.29–1.93	t = −3.359; p = 0.001
	N1/N2	41	0.14 ± 1.33	0.08 [−0.69–0.85]	−2.21–3.45	
Pathological stage	Stage II	37	−0.90 ± 1.39	−1.33 [−1.56–0.08]	−3.29–1.93	t = −3.359; p = 0.001
	Stage III	41	0.14 ± 1.33	0.08 [−0.69–0.85]	−2.21–3.45	
Histopathological and microenvironmental features						
Tumour budding	Bd1	33	−0.19 ± 1.37	−0.14 [−1.23–0.85]	−3.18–3.02	F = 1.253; p = 0.292
	Bd2	31	−0.66 ± 1.42	−1.12 [−1.49–0.13]	−2.85–3.45	
	Bd3	14	−0.04 ± 1.65	0.03 [−1.25–1.00]	−3.29–2.58	
Tumour-infiltrating lymphocytes	Low	45	−0.35 ± 1.53	−0.46 [−1.44–0.52]	−3.18–3.45	F = 0.004; p = 0.996
	Moderate	25	−0.35 ± 1.32	−0.36 [−1.44–0.73]	−2.21–1.93	
	High	8	−0.39 ± 1.53	0.02 [−1.06–0.85]	−3.29–1.06	

Abbreviations: SCD, stearoyl-CoA desaturase; PCNA, proliferating cell nuclear antigen; SD, standard deviation; Q1–Q3, first–third quartile; pN, pathological nodal stage; TILs, tumour-infiltrating lymphocytes; Bd, tumour budding grade; t, Student's t-test statistic; F, one-way ANOVA statistic. The analysis was restricted to moderately differentiated G2 colon adenocarcinomas with complete SCD IRS and PCNA IRS data.

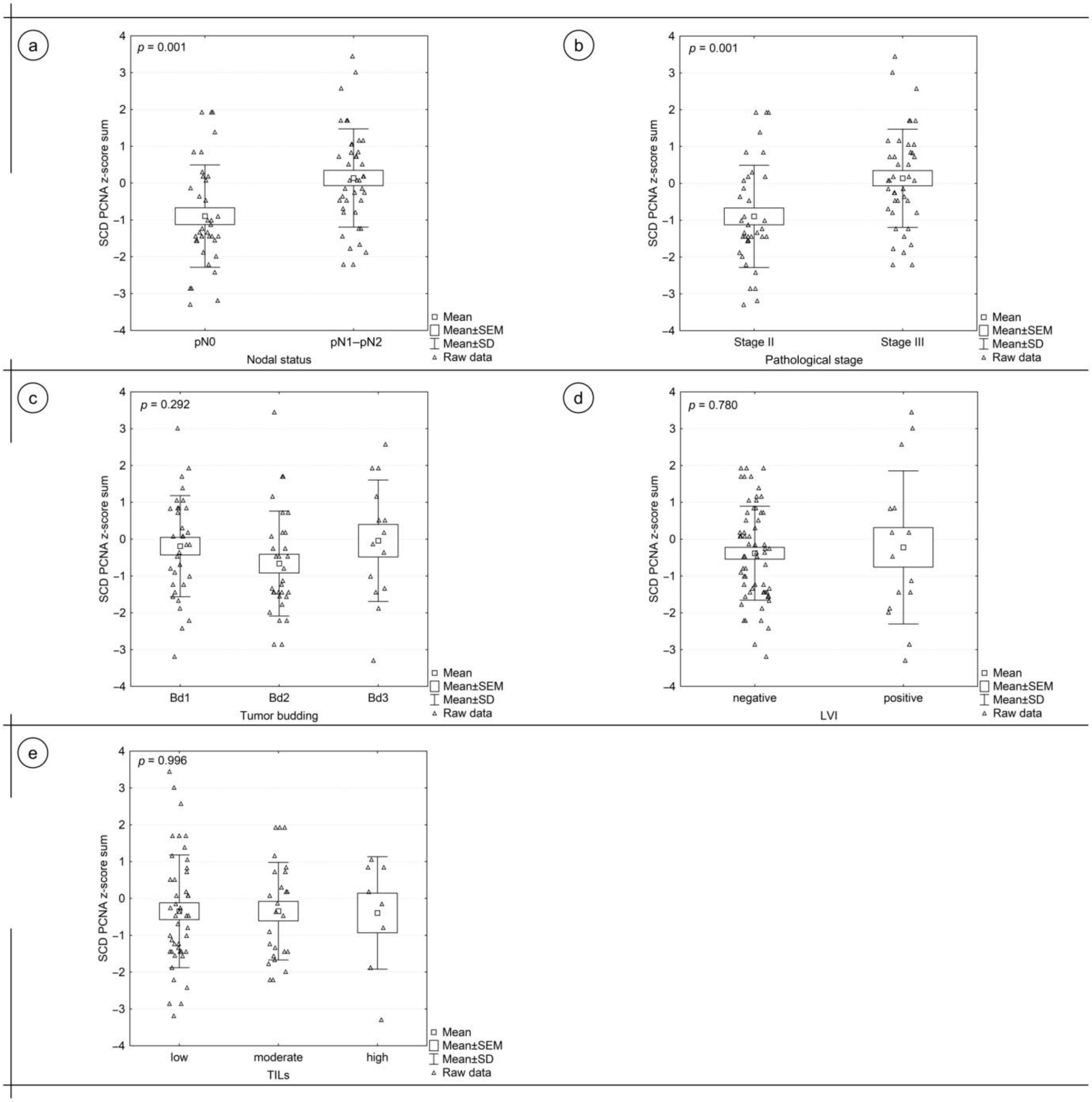

Supplementary Figure S2. Exploratory subgroup analysis of the combined SCD-PCNA z-score in moderately differentiated G2 colon adenocarcinomas. The analysis was descriptive and hypothesis-generating and was not intended to establish a clinical stratification approach. Panels show nodal status (a), pathological stage (b), tumour budding (c) and tumour-infiltrating lymphocyte density (d). The stage comparison is mathematically identical to the nodal-status comparison in this cohort. Squares indicate means, boxes indicate mean ± SEM, whiskers indicate mean ± SD, and triangles indicate individual raw data points.
