# Supplementary material for: Clinicopathological Significance of SCD Expression in Colon Adenocarcinoma: Association with Histological Grade and Exploratory Assessment in Relation to PCNA
Source: J Clin Med. 2026 Sep 2;15(17):6817. doi: 10.3390/jcm15176817 (PMC13566249; doi:10.3390/jcm15176817)
Supplement: Supplementary file 1 [file jcm-15-06817-s001.zip › Supplementary Material S1.pdf]

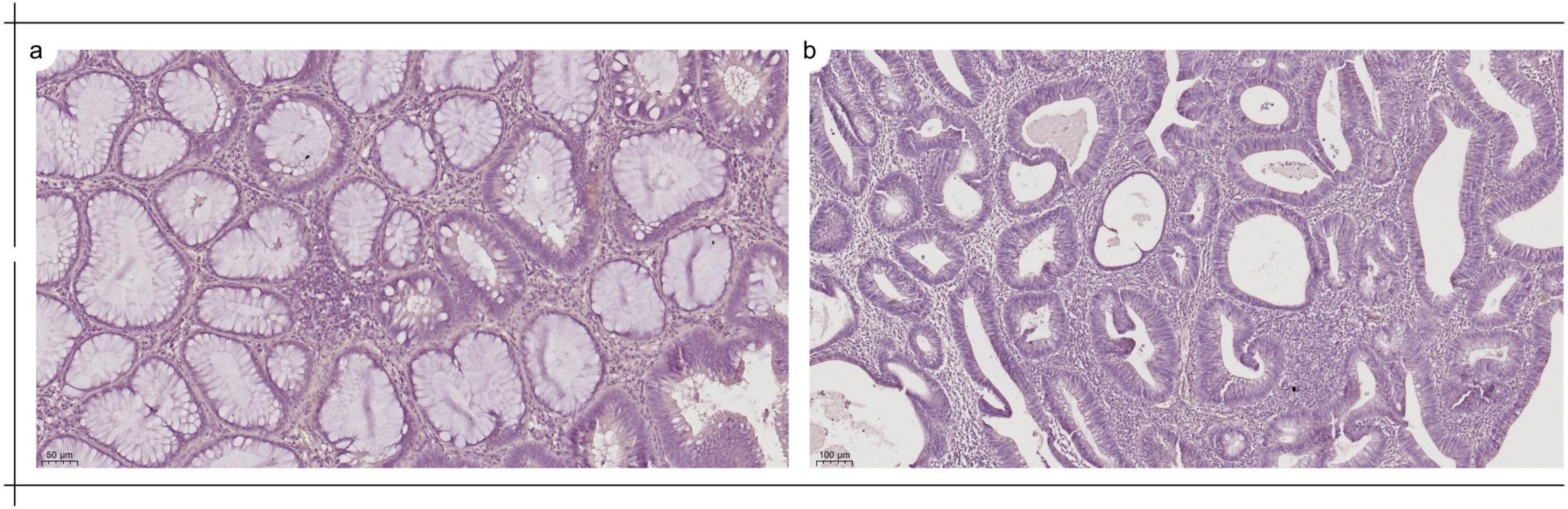

Figure S1 Negative controls for SCD immunohistochemical staining in non-neoplastic colonic mucosa and colon adenocarcinoma. Representative sections processed in parallel with omission of the primary anti-SCD antibody. (a) Non-neoplastic colonic mucosa and (b) colon adenocarcinoma tissue. No specific immunoreactive signal is observed, supporting the absence of nonspecific staining under the applied detection conditions. Scale bars: 50 µm in (a) and 100 µm in (b).
